# A Simple, Realistic Stochastic Model of Gastric Emptying

**DOI:** 10.1371/journal.pone.0153297

**Published:** 2016-04-08

**Authors:** Jiraphat Yokrattanasak, Andrea De Gaetano, Simona Panunzi, Pairote Satiracoo, Wayne M. Lawton, Yongwimon Lenbury

**Affiliations:** 1 Department of Mathematics, Mahidol University, Bangkok 10400, Thailand; 2 Center of Excellence in Mathematics, Bangkok 10400, Thailand; 3 Institute of System Analysis and Informatics (IASI) “A. Ruberti”, National Research Council (CNR), Rome, Italy; 4 School of Mathematics and Statistics, University of Western Australia, Perth, Australia; Montclair State University, UNITED STATES

## Abstract

Several models of Gastric Emptying (GE) have been employed in the past to represent the rate of delivery of stomach contents to the duodenum and jejunum. These models have all used a deterministic form (algebraic equations or ordinary differential equations), considering GE as a continuous, smooth process in time. However, GE is known to occur as a sequence of spurts, irregular both in size and in timing. Hence, we formulate a simple stochastic process model, able to represent the irregular decrements of gastric contents after a meal. The model is calibrated on existing literature data and provides consistent predictions of the observed variability in the emptying trajectories. This approach may be useful in metabolic modeling, since it describes well and explains the apparently heterogeneous GE experimental results in situations where common gastric mechanics across subjects would be expected.

## Introduction

Gastric Emptying (GE) is the process by which the stomach delivers food into the duodenum: the stomach exhibits regular churning contractions and these are occasionally coupled with peristaltic contractions and relaxation of the pyloric sphincter to produce intermittent squirting of partially digested chyme into the duodenum. GE is thus a highly coordinated physiological response to the presence of food in the stomach, which can be impaired in several pathological conditions [1–3]. Besides its intrinsic interest in relation to disturbances of Gastro-Intestinal (GI) motility, an understanding of GE is also very important for all those metabolic studies, which depend on the delivery of nutrients from the stomach into the absorbing portions of the GI tract [4–8]. It is now emerging that the rate of gastric emptying may be a major determinant of postprandial glycemic excursions in healthy subjects, as well as in Type 1 and Type 2 Diabetes Mellitus (T1DM, T2DM) patients: studies suggest that an inverse relationship between the rate of gastric emptying and blood glucose concentration exists in T2DM patients and that similar regulatory mechanisms may exist in both T1DM and T2DM [9].

It must be noticed that considerable intra-individual variability in gastric emptying rates has been observed in several studies.

Fraser and coAuthors [10] reported high intra-individual variability in stomach ethanol absorption. Twenty-four healthy subjects were studied in two different days. The ranges of time to peak plasma concentration were 16.1–51.1 min in the first study, and, 20.7–61.1 min in the second study. Furthermore, Fig 2 (page 389) shows the large differences between first and second study (in the same subjects) in the mean values of AUC, peak concentration and time to peak concentration.

**Fig 1:**
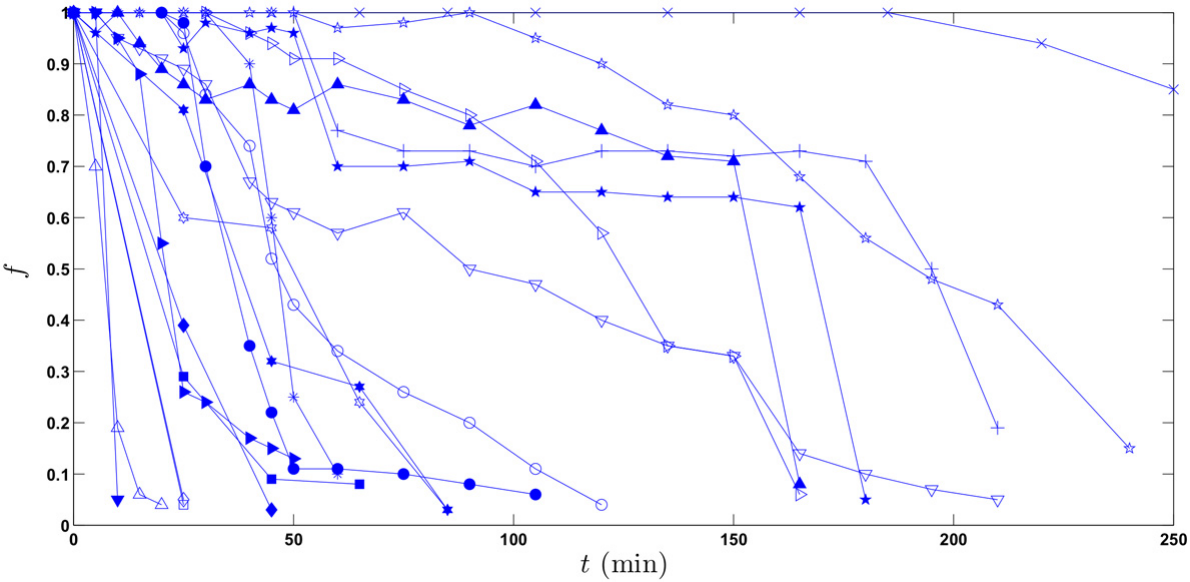
Connected plots of 19 scintigraphic data sets. Connected plots (solid lines) of 19 profiles acquired from Locatelli et al. [17]. Abscissa is time *t* after the pellet meal, ordinate is fraction *f* of meal remaining in the stomach at each time.

Pedersen [11] studied gastric emptying of a liquid meal. This Author reported that there was no evident relationship between the differences in twelve healthy subject pairs of repeated measurements (2 times at different days) and the corresponding mean values. As shown in Fig 1 on page 341, there were large fluctuations in sonographic antral area measurements before a meal of broth, immediately after the meal, and 10 min after the meal.

**Fig 2:**
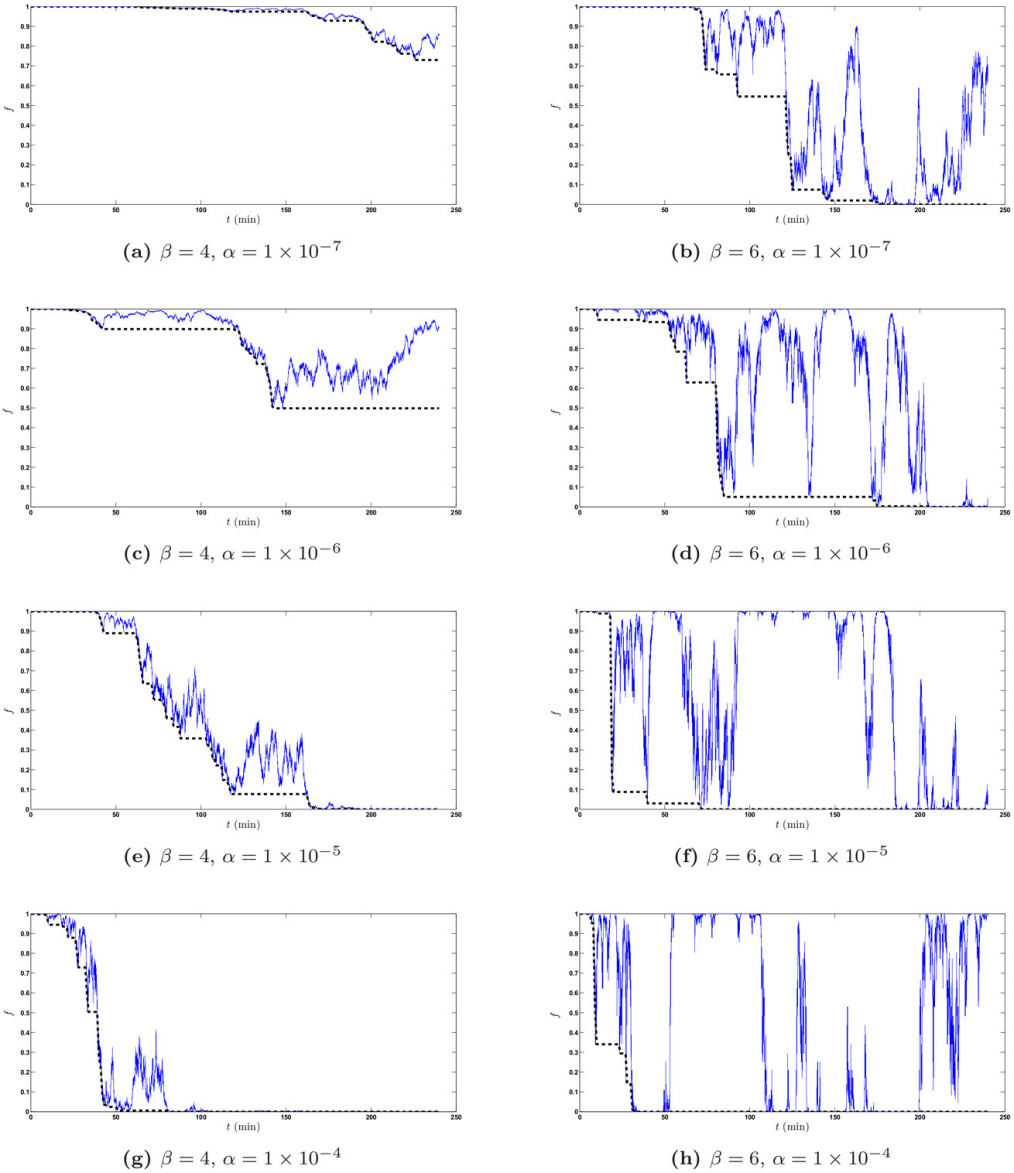
Model with the GE represented by Eqs 10 and 11. Model with the GE represented by Eq 10 (solid lines) and Eq 11 (dashed lines) in two different values of parameter *β*: *β* = 4 (2a, 2c, 2e and 2g) and *β* = 6 (2b, 2d, 2f and 2h). In each parameter *β*, *α* is differently considered: *α* = 1 × 10^−7^ (2a and 2b), *α* = 1 × 10^−6^ (2c and 2d), *α* = 1 × 10^−5^ (2e and 2f), and *α* = 1 × 10^−4^ (2g and 2h). Abscissa is time *t* after the pellet meal, ordinate is fraction *f* of meal remaining in the stomach at each time.

**Fig 3:**
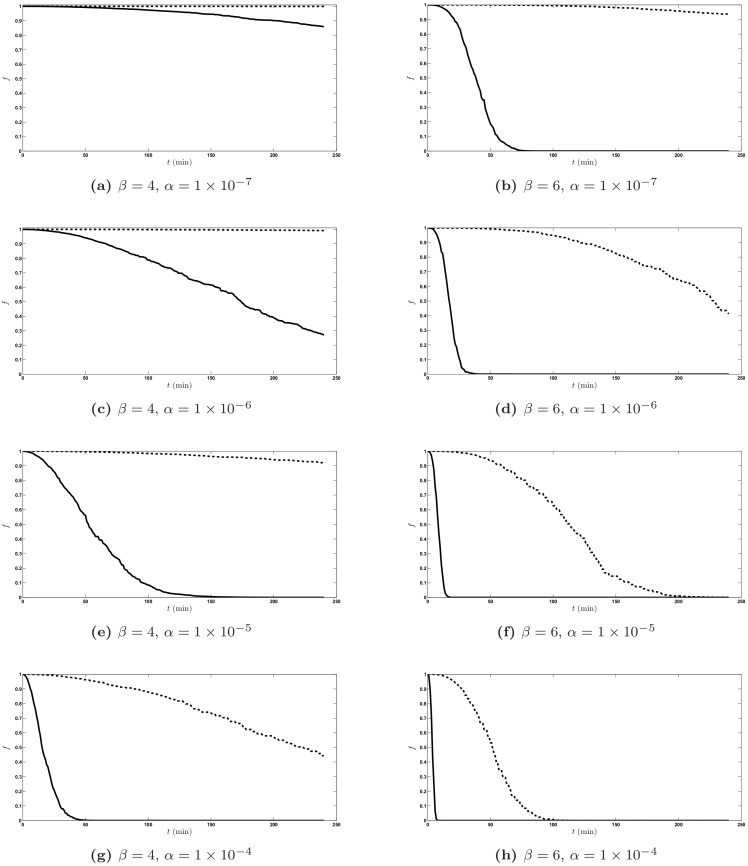
90% two-sided confidence bounds of *S*(*t*). 90% two-sided confidence bounds of *S*(*t*): the 5th percentile (solid lines) and the 95th percentile (dashed lines). Parameters: *β* = 4 (3a, 3c, 3e and 3g), *β* = 6 (3b, 3d, 3f and 3h). *α* = 1 × 10^−7^ (3a and 3b), *α* = 1 × 10^−6^ (3c and 3d), *α* = 1 × 10^−5^ (3e and 3f), and *α* = 1 × 10^−4^ (3g and 3h). Abscissa is time *t* after the pellet meal, ordinate is fraction *f* of meal remaining in the stomach at each time.

Brophy and coAuthors [12] reported high intrasubject variability for both the emptying of solids and the emptying of liquids using traced meals. Eight healthy subjects were studied four times on different days. As shown in Figs 2 and 3 on page 803, there were wide ranges in liquid and solid half emptying times, respectively, within and between subjects.

In fact, the process of gastric emptying is far from continuous. Several concurrent, partially coordinated mechanisms, contribute to the mixing of stomach contents; to the formation of peristaltic waves and of retrograde waves (retropulsion); to the temporary limited opening of the pyloric sphincter; and to the evidential squirting ejection of sufficiently liquid chime [13–16]. While approximate frequencies of chyme expulsion from the stomach are assessed in experimental preparations, during each meal the moments at which squirting through the pylorus occurs as well as the corresponding squirted chyme amounts are irregular.

Several models have been proposed to describe the rate of GE (for both liquid and solid meals) over time, in humans and in animals. These include at least mono-exponential models [6, 8]; a lag-time exponential model [17]; power exponential models [4, 7, 17–19]; a double power exponential model [17]; modified power exponential models [20, 21]; and nonlinear first order deterministic elimination models [22]. These models are summarized below.

**Mono-exponential models:** Ogungbenro and Aarons [6] proposed a semi-mechanistic model for analyzing ^13^C-ocanoic acid breath test (a measurement of GE) data. The model had five compartments. In the stomach compartment, a mono-exponential function

$$y\left(t\right)=e^{-kt}$$ (1)

was applied, where *y*(*t*) is the fraction of the liquid meal retained in the stomach at time *t*; and *k* is the GE rate.

Yu and Amidon [8] modeled a compartmental absorption and transit system in the gastrointestinal tract for estimating oral drug absorption. As a part of the overall model, the delivery of drug from the stomach into the small intestine was represented as first-order deterministic elimination:

$$\frac{dy}{dt}=-ky,$$ (2)

where *y*(*t*) is the amount of drug in the stomach; and *k* is the emptying rate constant. From this equation the gastric drug retention is directly computed as:

$$y\left(t\right)=Ce^{-kt},$$ (3)

where *C* is a constant expressing initial gastric drug content. This equation clearly corresponds to Eq (1) above.

**A lag-time exponential model:** Locatelli et al. [17] proposed three mathematical models that describe human GE of pellets under fasting conditions. One of these models is a lag-time exponential model

$$y\left(t\right)\left[\%\right]=100e^{-k(t-t_{lag})},$$ (4)

where *y*(*t*) is the proportion of pellets still remaining in the stomach at time *t*; *k* is the first order elimination rate constant; and *t*_*lag*_ is the delay time in the GE. The other two Locatelli models are described below.

**Power exponential models:** Locatelli et al. [17] proposed a second GE model incorporating a two-parameter Weibull distribution over time:

$$y\left(t\right)\left[\%\right]=100e^{-{\left(\frac{t}{\eta }\right)}^{\beta }},$$ (5)

where *y*(*t*) is the proportion of pellets still remaining in the stomach at time *t*; *η* is the scatter parameter; and *β* is the shape parameter.

Salinari et al. [7] proposed a mathematical model for the intestinal transit of a glucose bolus. They described GE as a power exponential function

$$y\left(t\right)=e^{-{\left(kt\right)}^{\beta }},$$ (6)

where *y*(*t*) is the fraction of glucose retained in the stomach at time *t* after glucose ingestion; and *k*, *β* are constants.

**A double power exponential model:** Again Locatelli et al. [17] proposed a third GE model resulting from the sum of two of the previous Weibull models:

$$y\left(t\right)\left[\%\right]=\left(100-H\right)e^{-{\left(\frac{t}{\eta_{1}}\right)}^{\beta_{1}}}+He^{-{\left(\frac{t}{\eta_{2}}\right)}^{\beta_{2}}},$$ (7)

where *y*(*t*) is the proportion of pellets still remaining in the stomach at time *t*; *η*_1_, *η*_2_ are the scatter parameters; *β*_1_, *β*_2_ are the shape parameters; and *H* is the percentage of pellets remaining in the stomach when GE has temporarily stopped. When assessing their three models, Locatelli et al. [17] concluded, on the basis of the Akaike criterion, that this sum-of-Weibulls best fitted individual published data.

**A modified power exponential model:** Siegel et al. [21] used a modified power exponential function

$$y\left(t\right)=1-{\left(1-e^{-kt}\right)}^{\beta },$$ (8)

where *y*(*t*) is the fractional meal retention at time *t*; *k* is the gastric emptying rate; and *β* is the extrapolated *y*-intercept from the terminal portion of the curve. The goal of these Authors was to obtain fractional meal retention values for analyzing the characterization and quantification of the lag phase and the GE rate for both solids and liquids.

**A nonlinear first order deterministic elimination model:** Stubbs [22] applied the laws of Laplace, Hooke, and Poisseuille to derive an equation of GE for human adults

$$\frac{dy}{dt}=-ky^{p}\left(1-{\left(\frac{v}{y}\right)}^{n}\right),$$ (9)

valid when *y*(0) < 300 ml. Here *y*(*t*) is the volume in the stomach at a given time *t* after a meal; *k* is the emptying rate constant, related to the composition of the meal; *v* is the volume of a resting stomach; and *p*, *n* are constants. By minimizing the squared residuals between some published data and the fitted values obtained from the equation, they estimated $p=\frac{4}{3},n=\frac{1}{2}$ and *v* to about 28 ml.

In our view, the essential limitation of all of the above models is that they describe GE as a continuous, smooth process in time. We however know that GE is a discontinuous process [23–26], which proceeds in spurts separated by quiescent periods and that both the timing and the volume of the spurts appear random to the observer. The aim of the present study is therefore to formulate a stochastic model, which can describe this behavior and which can be used in the future to model gastrointestinal tract function.

Stochastic models have already been proposed, in alternative to deterministic models, in other areas of biomedical research [27–30]. In all of these cases the rationale being that deterministic models fail to correctly capture system behavior where conditions are far from the usual assumptions for mean-field approximations. In the present case as well, using an exponential or other similar deterministic model would lead to incorrect conclusions if the underlying system were inherently stochastic. These considerations motivate the search for a simple, plausible model, qualitatively coherent with known physiology.

## Materials and Methods

### The model

A stochastic model for gastric emptying is desired, such that at time *t*_0_ the (fractional) residual meal content of the stomach is 1 (i.e. 100% of the meal, supposed to be instantaneously delivered to the stomach at time *t*_0_), and such that, by successive random (instantaneous) “spurts” the residual content of the stomach decreases towards zero. In order to formulate this first stochastic gastric emptying model (S-GEM1), we start with a standard Wiener process *W* = {*W*(*t*)∣*t* ≥ 0}. Next, the power exponential function of standard Wiener process is evaluated as:

$$X\left(t\right)=e^{-\alpha {\left|W(t)\right|}^{\beta }},$$ (10)

where *α* and *β* are parameters determining the shape of the variation of *X* over the positive reals. Finally, the fractional residual meal content (*S*(*t*)) at a given time *t* can be represented as

$$S\left(t\right)=\min_{0<s<t}X\left(s\right).$$ (11)

The meaning and units of measurement of the model’s state variables and parameters are reported in Table 1.

**Table 1:** State variables and all parameters in the S-GEM1 model.

Symbol	Extended Name	Value	Unit
*t*	Time	-	minute
*W*	Standard Wiener process	-	#
*X*	Power exponential function of Wiener process	-	#
*S*	Fractional residual meal jump process	-	#
*α*	Parameter	1 × 10^−6^	#
*β*	Parameter	6	#
${\left\{t_{j},\;u_{j}\right\}}_{j=1}^{\infty }$	Actual spurts	-	(minute, #)

Clearly, *S*(*t*) will be a monotonically non-increasing jump process, where both the timing and the size of the jumps are random and whose overall behavior roughly resembles a decreasing exponential, given that large downward jumps of *X*(*t*) typically appear at earlier times.

Notice that the actual spurts are given by the sequence ${\left\{t_{j},\;u_{j}\right\}}_{j=1}^{\infty }$, where {*t*_*j*_} = {*t* ∣ *S*(*t*^+^) < *S*(*t*)} and $\left\{u_{j}\}=\{S\left(t_{j}\right)-S\left(t_{j}^{+}\right)\right\}$.

As an example, S-GEM1 could be used, within a broader model of the glucose-insulin system, by incorporating the resulting sequence of times and amounts of glucose entry into the jejunum, for instance, as

$$\frac{dJ}{dt}=-k_{GJ}J+M_{0}\sum_{j=1}^{\infty }\delta \left(t-t_{j}\right)u_{j}$$ (12.1)

$$\frac{dG}{dt}=-k_{XGI}GI+k_{\xi }+k_{GJ}J+\ldots \;$$ (12.2)

$$\frac{dI}{dt}=\ldots \ldots \ldots \ldots$$ (12.3)

………………, (……)

where J is the amount of glucose in the jejunum while *G* and *I* respectively represent glucose and insulin plasma concentrations and *M*_0_ is the total amount of glucose in the meal. Notice that Eqs (12) above are deterministic except for the random set of times and amounts {*t*_*j*_, *u*_*j*_}.

### Data acquisition

In order to adapt our model to real data, we graphically acquired the 19 individual data sets presented in Locatelli et al. (see S1 File), referring to human GE studies conducted by means of gamma-scintigraphic measurement of residual stomach content of orally administered pellets under otherwise fasting conditions.

The nineteen scintigraphic data sets in Locatelli et al. [17] were all taken from young healthy subjects, most of them male. The pellet size given to the subjects varied between 0.5 and 5 mm; pellets were accompanied with 100–200 ml of water or orange juice. During the evaluation of GE, all subjects were studied in the upright body position.

### Simulation study

Each individual was simulated over 240 minutes, with a discretization step Δ*t* = 0.01 min.

The 90%two-sided path confidence bounds for each couple of *α* and *β* parameter values were evaluated numerically over a sample of 2000 trajectories for each parameter combination.

No formal parameter estimation was carried out, both for simplicity and because the original data were not available to us (only graphically acquired points from a published paper were used). Instead, the parameter combination was chosen, from all those used for simulation, which generated a 90% confidence envelope broadly consistent with the reported Locatelli’s data [17].

## Results

For each couple of parameter values (*α* and *β*) 2000 trajectories were obtained from the model, corresponding to 2000 stochastic realizations of the same emptying process (as defined by the generating parameter values). What is not commonly appreciated in the medical environment is that observed profiles corresponding to identically structured physiological phenomena can appear strikingly different, when system noise is non-negligible. In the simulations obtained, in fact, there is often a very considerable spread in the 2000 trajectories obtained with identical parameter values. This spread is obviously greater when neither the *α* nor the *β* parameter are either very large or very small. When either parameter is very large the probability of rapid emptying becomes so large that all trajectories are grouped in the bottom-left region of the time-content plane. When both parameters are very small, stochastic variability as well as emptying rate are minimal, and trajectories are grouped at the top of the time-content plane. These findings are wholly expected and consistent with the physiology: substantial variability being expected in normal subjects, while in paralyzed stomach as well as in, say, retching (which we mention for the sake of argument, even though it does not represent a case of forward gastric emptying), the expected behavior is much the same in all affected subjects.

Fig 1 shows the nineteen graphically acquired original subjects from Locatelli et al. [17], each connected sequence corresponding to a different subject.

Fig 2 shows eight examples of individual simulated GE profiles from the stochastic model.

For each Fig 2 panel, both the *X* process (continuous line) and the corresponding (downward) jump process *S* (dashed line) are shown.

The left panels of Fig 2 report examples for *β* = 4, the right ones examples for *β* = 6. In each column, the four panels correspond, top to bottom, to increasing values of *α* (1 × 10^−7^, 1 × 10^−6^, 1 × 10^−5^ and 1 × 10^−4^).

It can be appreciated that when the volatility of *X* is very large (e.g. panels 2d, 2f, 2h), the *S* process falls to zero very quickly. Conversely, when the volatility of *X* is small, *S* falls slowly (e.g. panel 2c) or very slowly (e.g. panel 2a).

Fig 3 shows the two-sided confidence bounds of trajectories obtained with different combinations of *α* and *β* parameter values. The panels in Fig 3 are arranged in the same way as the panels in Fig 2, the left column corresponding to *β* = 4, the right column to *β* = 6, and panels within each column corresponding, top to bottom, to *α* = 1 × 10^−7^, 1 × 10^−6^, 1 × 10^−5^ and 1 × 10^−4^.

An increase in *β* corresponds to a more rapid gastric emptying at any level of *α*, and similarly an increase of *α* also corresponds to a faster overall rate of gastric emptying. Varying the one or the other parameter however produces different changes in the shape of the 90% trajectory envelope.

**Fig 4:**
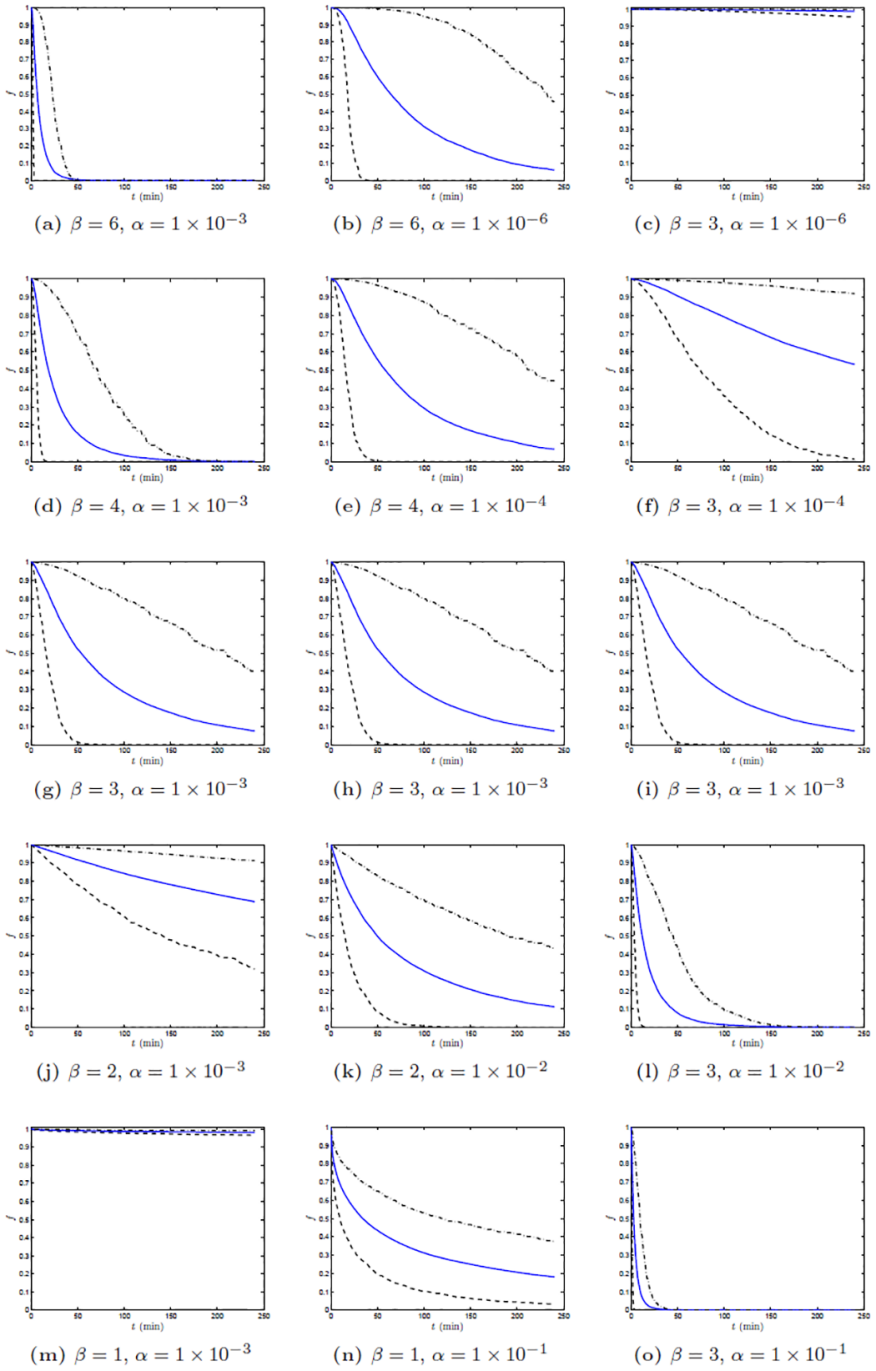
90% two-sided confidence bounds and mean of *S*(*t*). 90% two-sided confidence bounds and mean of *S*(*t*): the 5th percentile (dashed lines), the 95th percentile (dashed-dot lines) and the mean (solid lines). Parameters: *β* = 6 (4a and 4b), *β* = 4 (4d and 4e), *β* = 3 (4c, 4f, 4g, 4h, 4i, 4l and 4o), *β* = 2 (4j and 4k), *β* = 1 (4m and 4n). *α* = 1 × 10^−6^ (4b and 4c), *α* = 1 × 10^−4^ (4e and 4f), *α* = 1 × 10^−3^ (4a, 4d, 4g, 4h, 4i, 4j and 4m), *α* = 1 × 10^−2^ (4k and 4l), and *α* = 1 × 10^−1^ (4n and 4o). Abscissa is time *t* after the pellet meal, ordinate is fraction *f* of meal remaining in the stomach at each time.

**Fig 5:**
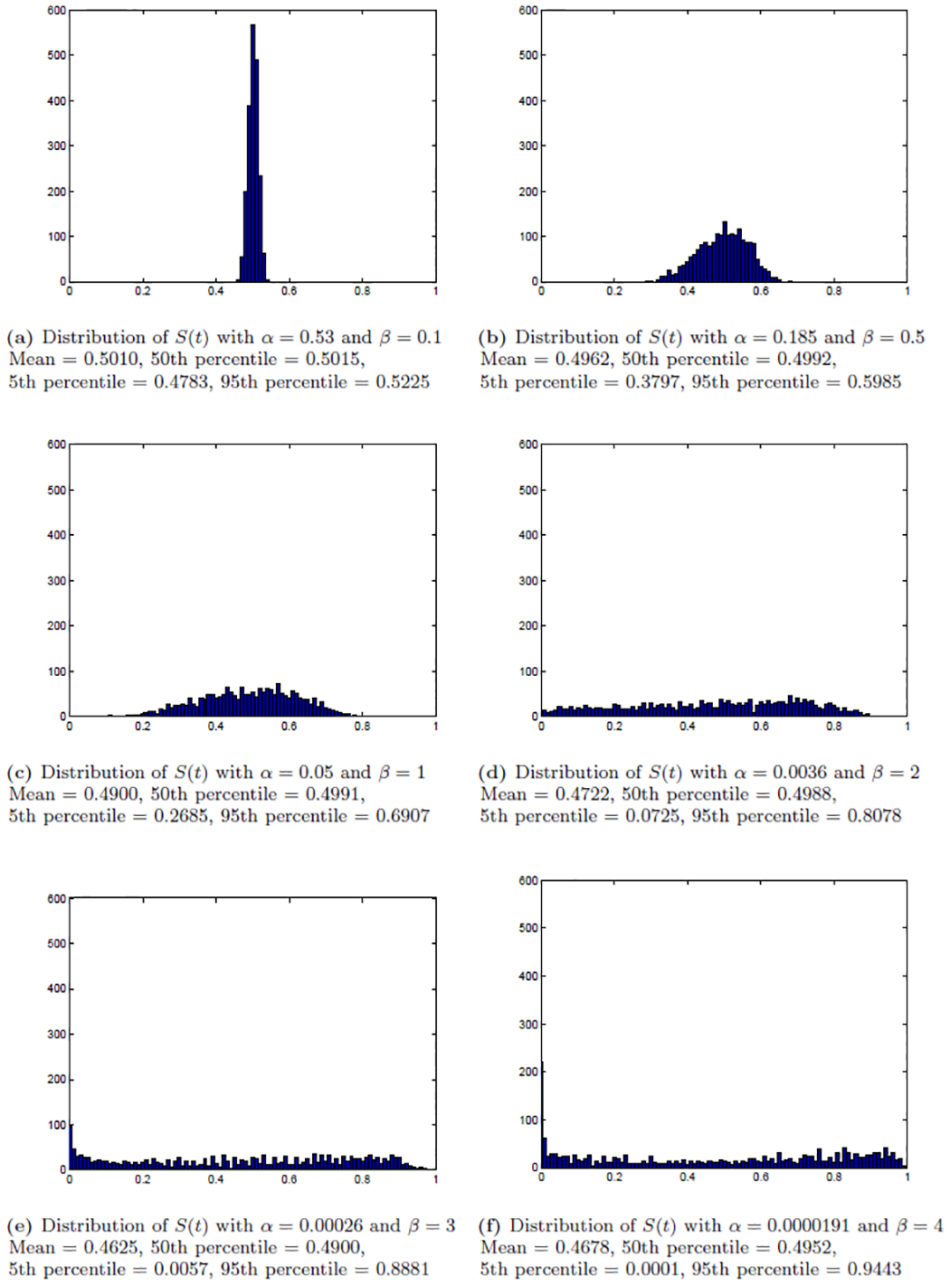
Comparison of the distributions of the jump process *S*(*t*) at *t* = 150 min. The distributions of *S*(*t*) from 2,000 simulations at *t* = 150 min corresponding to different sets of parameter values, each of which has the 50th percentile of the process *S*(*t*) approximately equal to 0.5. Abscissa is the values of *S*(*t*) at *t* = 150 min and ordinate is the frequency.

Fig 4 shows the mean, 5th and 95th percentile of 2000 trajectories of *S*(*t*). Fig 5 shows the distribution of the 2000 values of *S*(*t*) at *t* = 150 minutes for various combinations of parameter values.

In Fig 4, in the left column, an increase in *β* corresponds to a more rapid gastric emptying, and similarly in the right column, an increase of *α* also corresponds to a faster overall rate of gastric emptying. Varying the one or the other parameter however produces different changes in the shape of the 90% trajectory envelope. It should be emphasized that in order to maintain the overall rate of gastric emptying for different choices of parameter values, if one increases the values of *β*, then one needs to decrease the values of *α*. As a result, the 90% trajectory envelope will be wider in the top panels and tighter in the bottom panels (as shown in the middle column). The different behavior of increasing one or the other parameters can also be examined by comparing the distributions of the jump process *S* at some fixed time, e.g. at *t* = 150 min, for various combinations of parameter values. For each panel in Fig 5 the 50th percentile of the process *S* at *t* = 150 min is approximately equal to 0.5, i.e. approximately half of the trajectories show less than 0.5 emptying at 150 minutes and half show larger than 0.5 emptying at 150 minutes, or, in other words, at the fixed time 150 minutes 50 percent of the realizations have less than one half the initial contents and the other 50 percent have more than one half the initial contents. Each panel in Fig 5 corresponds to a different combination of values of *β* and *α*, but for all these combinations the median at 150 minutes is always 0.5. However, for smaller values of *β* and larger values of *α* (e.g. panel a), the distribution of *S*(150) is tightly concentrated around its median, while as the values of *β* increase and the values of *α* decrease, the distribution of *S*(150) becomes more and more dispersed (e.g. panels e and f), corresponding to a large variability of emptying at this time among realizations obtained with the same parameter values.

**Fig 6:**
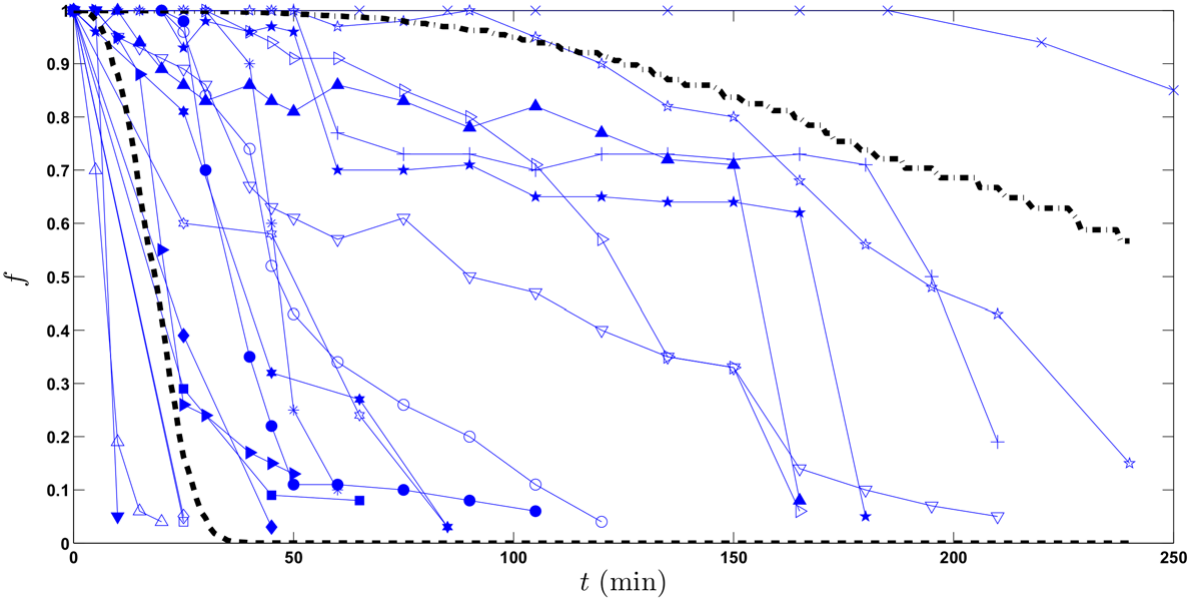
Connected plots of 19 profiles together with 90% two-sided confidence bounds of *S*(*t*). Connected plots (solid lines) of 19 profiles acquired from Locatelli et al. [17] together with 90% two-sided confidence bounds of *S*(*t*): the 5th percentile (dashed line) and the 95th percentile (dash-dot line) corresponding to parameters *α* = 1 × 10^−6^ and *β* = 6. Abscissa is time *t* after the pellet meal, ordinate is fraction *f* of meal remaining in the stomach at each time.

Fig 6 shows again the graphically acquired data from the original Locatelli paper [17] together with the 90% confidence bound, obtained numerically pointwise in time from 2000 realizations of the S-GEM1 solutions with parameters *α* = 1 × 10^−6^ and *β* = 6.

It should be noted that 9.73% of the Locatelli observations, obtained from six of the observed patients fell outside this theoretical 90% confidence band.

## Discussion

As is apparent from the brief review of previous mathematical models for GE reported in the Introduction, rates of GE are generally calculated by fitting residual gastric content observations to deterministic mathematical representations of the emptying process. Several such GE models have been proposed so far, based on exponential, power exponential, double power exponential, linear-exponential, modified power exponential and deterministic elimination models [4, 6–8, 17–22]. Currently, the most popular models for both liquid and solid meals are the exponential and power exponential models [4, 6–8, 17–19].

As has already been mentioned before, however, the actual process of GE is characterized physiologically by a random sequence of spurts of semiliquid, semidigested boli through the pylorus into the duodenum. This sequence of spurts is apparently random both in timing and in size (volume of the boli), with the evident constraint that, as the stomach empties, the boli must eventually decrease in size. Any deterministic model of the above process is a rather crude approximation, whose value for applications mainly resides in its simplicity. The availability of a reasonably simple stochastic model for GE could be important for applications if it allowed a better representation of the inherent variability of the GE process.

Stochastic models in literature are very frequent and they are used in different fields: in finance (interest rate, stock prices), biology (population dynamics, epidemics), physics (fluid particles thermal noise), control and signal processing (controllers, filtering). When modeling the time course of a variable of interest as a stochastic process, different approaches can be followed: it is possible to define a stochastic differential equation for the variable, or to define an equation where the time evolution of the stochastic variable is directly represented, or to express the time evolution of the probability density function of the variable. In the present work, we propose a formulation of the relevant stochastic system by directly specifying the evolution of the stochastic variable ‘Residual Stomach Content’ over time, as driven by a Wiener process. Generally speaking Wiener processes with drift are often applied to modelling practical situations in which deterministic processes are disturbed by random fluctuations. Examples of such an approach are given by Wiener-based degradation models, used to characterize the path of degradation processes where the degradation increases linearly over time and where fluctuations in degradation can be observed [31–33]. In this kind of approaches the main interest is in Remaining Useful Lifetime (RUL) prediction.

Other examples are the exponential Levy models, frequently used in finance, which generalize the classical Black and Scholes models by allowing the stock prices to jump while preserving the independence and stationarity of returns. The introduction of a Brownian motion with drift in these kinds of models allows for continuous trajectories: it is interesting to note that an exponential Levy model can be defined both starting from a differential equation model or directly from its exponential formulation.

The most widely used approach to describe continuous time processes is however that of extending the classical ordinary differential equation models by taking into account the variability in the dynamics of the system, using Stochastic Differential Equations (SDE) models, which allow for the explicit representation of both intrinsic dynamical system noise together with observation error [34–39]. Classical, well established examples of SDE models are found in finance for the study of the behavior of the prices of financial assets (e.g. stocks, bonds, currencies, commodities). In biomedicine, SDE models have not been frequently used so far. There have however already been examples of application of SDE models to some specific research areas such as pharmacokinetic/pharmacodynamic (PK/PD) [40–44], cancer [34], neuronal firing [38] and metabolism [37, 45, 46].

In the present work the approach followed was that of directly defining an equation for the time evolution of the fractional residual meal content in the stomach; a simple stochastic process model is proposed to address the intrinsically noisy behavior of the GE mechanism.

The proposed model satisfies the requirements expressed in the introduction: it is a simple stochastic model, it mimics physiology well, and it can usefully reproduce actual observations.

We see that, in the proposed model, there are two parameters *α* and *β* whose combined effect is to vary the shape of the 90% confidence region of the solution paths. In particular, when either *α* or *β* increases, GE trajectories are on the average faster.

Further, the reason of developing the proposed model instead of writing a stochastic differential equation (SDE) is because it is not readily apparent what physiology-based SDE would have solutions *S*(*t*,*W*) which are strictly positive and bounded 0 ≤ *S*(*t*,*W*) ≤ 1 ∀*t*,*W*, non-increasing, constant over $R^{+}\backslash A$ where *A* is the set of no spurts in gastric emptying, and $\lim_{t\to \infty }S\left(t,W\right)=0$ ∀*W*. In fact, the above criteria define the behavior of stomach emptying from a physiological point of view.

One limitation of the current model is that it considers a homogeneous meal. It is well known, however, that after a usual meal composed of both liquids and solid foodstuffs, the stomach quickly empties itself of liquids, while solids take a longer time to be digested. Similarly, fatty meals take a longer time being digested than non-fat-containing meals. Future versions of the model may therefore contemplate different stochastic processes, corresponding to foods of different mechanical and chemical characteristic.

Another limitation of the present study is in fact that no formal parameter estimation procedure has been performed on the available data. The reason is that multi-level, mixed-effects procedures for stochastic process models are indeed rather cumbersome, and that the main concern of the present work is the formulation and physiological justification of the model. Parameter estimation will need to be addressed with future work.

It must be noticed that to the same couple of parameter values for *α* and *β* (for instance, *α* = 1 × 10^−6^, *β* = 6, see Fig 3 panel d) there corresponds a very marked variability of solution paths. This behavior, which is typical of the solutions of stochastic differential equations, is usually surprising to the medical practitioner. In fact, the choice of parameter values (*α* = 1 × 10^−6^, *β* = 6) corresponds very well to the observed variability in the trajectories actually observed by Locatelli et al. [17], whose subjects could therefore belong to a homogeneous group in terms of gastric emptying mechanics. This interpretation, which is supported by the actual model simulations, would typically be in contrast with a mere inspective appraisal of Locatelli’s data, data which would seem apparently consistent with a whole wide range of GE behaviors. In fact, the original observations were obtained by Locatelli et al. [17] from a relatively homogeneous group of subjects: young, healthy, mostly male, in nearly identical experimental conditions. The fact that a single set of parameter values can describe apparently diverse GE results is therefore very plausible if we consider the underlying subjects’ physiology.

It is a matter of direct, concrete, practical interest in the analysis of Locatelli’s original observations to decide whether a deterministic model is sufficient or if a stochastic process model needs to be used. In fact, if we used a deterministic model (say, an exponential decay), we would in all likelihood conclude that the original sample of subjects is heterogeneous with regards to GE, and we would consequently be led to investigate the medical determinants of this heterogeneity (disease, body habitus, lifestyle, etc.). Conversely, if we used the stochastic process model proposed in the present work, we would in all likelihood conclude that the apparently heterogeneous GE experiments are entirely consistent with common, uniform gastric mechanics characteristics, the apparent heterogeneity being well explained by the inherent variability in the emptying process itself. The decision of using deterministic or stochastic dynamical models makes therefore a clear difference in the interpretation of the biology and in the conclusions which can be drawn from the experiments.

In conclusion, a simple stochastic process model of GE can be formulated, is physiologically very plausible, can be easily incorporated into more complex metabolic models, and can provide new insights into the results of classical experiments.

## Supporting Information

S1 File Gastric emptying of pellets under fasting conditions: a mathematical model. doi: 10.1007/s11095-009-9869-3.
(PDF)
