# Supplementary material for: A Simple, Realistic Stochastic Model of Gastric Emptying
Source: PLoS One. 2016 Apr 8;11(4):e0153297. doi: 10.1371/journal.pone.0153297 (PMC4825969; doi:10.1371/journal.pone.0153297)
Supplement: S1 File — doi: 10.1007/s11095-009-9869-3. (PDF) [file pone.0153297.s001.pdf]

Pharmaceutical Research, Vol. 26, No. 7, July 2009 (© 2009)
DOI: 10.1007/s11095-009-9869-3

Research Paper

Gastric Emptying of Pellets under Fasting Conditions: A Mathematical Model

Igor Locatelli,[1] Aleš Mrhar,[1] and Marija Bogataj[1,2]

Received August 21, 2008; accepted March 2, 2009; published online April 1, 2009

Purpose. To develop a mathematical model that would adequately describe human gastric emptying of pellets under fasting conditions of healthy subjects.
Methods. Scintigraphic profiles representing the gastric emptying of pellets were obtained from the literature. Altogether 19 individual and three mean scintigraphic profiles were collected. Three mathematical models namely; the lag-time exponential (two parameters), the Weibull (two parameters), and the double Weibull (five parameters) model were proposed and fitted to the gastric emptying profiles.
Results. Different patterns of gastric emptying (immediate and rapid, delayed but rapid, delayed and slow, and interruptive emptying) were observed, with the emptying time varied from approximately 15 min to more than 3 h. The best model for fitting to the individual profiles was the double Weibull model. This model also provided an insight into the mechanism of interruptive emptying of pellets, observed for some patients. In addition, mean gastric emptying of pellets was calculated using the Weibull model.
Conclusions. Mean gastric emptying of pellets was adequately described by the Weibull model (η= 61.9 min, β=0.895), which could be applied in the design of *in vitro* dissolution experiments for pellet formulations with pH dependent dissolution.

KEY WORDS: drug dissolution; gastric emptying; mathematical models; multiparticulate systems; pellets.

INTRODUCTION

Gastrointestinal transit of oral dosage forms is most widely evaluated by a non-invasive technique called gamma scintigraphy (1). Recently, oral dosage forms have become more and more sophisticated, meaning that the transit characteristics of these forms through the gastrointestinal tract might have a more important impact on drug bioavailability, and could, therefore, largely contribute to inter- and intraindividual variability of the pharmacokinetic properties of a drug.

This paper is focused on the kinetics of gastric emptying, a crucial factor in the gastrointestinal transit of oral dosage forms. It is known that gastric emptying is influenced by various physiological and pathological factors, drug delivery system properties, and most of all, by food intake (2,3). Especially for multiparticulate formulations (e.g. pellets), the kinetics of gastric emptying in individual subjects is complex and very variable and thus difficult to evaluate (3). However, emptying of the stomach under fasting conditions is closely subjected to the migrating motor complex (MMC). In this view, the short lasting intense contractions that represent phase III of approximately 2 h long MMC cycle are the cause of effective emptying of the stomach contents in the fasted state (4).

So far, two mathematical models have been used to analyze the gastric emptying of multiparticulate formulations (5). The first, an exponential model with a lag-time or a "starting index", describing the time until the start of gastric emptying, was fitted to the profiles, where a delay in emptying occurred. However, a power exponential model was shown to be superior for fitting the gastric emptying data. In this model, the power parameter β determined the shape of various emptying profiles (e.g. a mono- or bi-exponential profile). Moreover, it was also stressed that this model should be fitted using nonlinear least squares regression rather than by linearization of the model function (5).

The gastric transit time of oral dosage forms is also important for release of drugs and consequently for their bioavailability. If drug release from the dosage form is pH dependent, then its release profile changes with gastric residence time. And in the case of pellets, which usually are not emptied from the stomach all at the same time, the fractions of pellets which remain in the stomach for different periods of time might release the drug with different kinetics. Therefore, it is very important to know the kinetics of gastric emptying of pellets, thus enabling the calculation of how long a certain fraction of pellets remains in the stomach. On this basis, it is possible to design an *in vitro* dissolution test and to assign the time periods during which pellets have to be kept in artificial gastric juice.

[1] Faculty of Pharmacy, University of Ljubljana, Aškerčeva cesta 7, 1000 Ljubljana, Slovenia.
[2] To whom correspondence should be addressed. (e-mail: marija.bogataj@ffa.uni-lj.si)

0724-8741/09/0700-1607/0 © 2009 Springer Science + Business Media, LLC

The objective of this study was to develop a mathematical model that would adequately describe human gastric emptying of pellets under fasting conditions of healthy subjects. By modeling the gastric emptying of pellets an additional insight into the mechanism of gastric emptying of pellets could be given. Furthermore, by using most descriptive model a mean gastric emptying profile was aimed to be generated. In our further work it will be used for determination of the experimental conditions of *in vitro* dissolution tests for pellet formulations with pH dependent dissolution.

MATERIALS AND METHODS

Data Acquisition

A systematic literature search on evaluation of human gastric emptying of pellets based on the technique of gamma scintigraphy was undertaken. Only those papers where fasting conditions in healthy subjects were assured in the study design were selected. Additionally, special attention was paid to the following criteria: pellet size and density, fluid intake with the administration of pellets, provision of refreshments after the administration, and subject position during imaging.

Graphical and numerical data representing the gastric emptying of pellets were evaluated and collected from the papers fulfilling the above criteria. Numerical data were represented by the individual values of the emptying parameters such as t_{50} (time when 50% of the administered pellets remained in the stomach), MGRT (mean gastric residence time), AUC (area under the emptying curve), and t_{lag} (time when pellets started emptying from the stomach). Individual and mean scintigraphic profiles were read out from the figures presented in the selected papers.

Statistical Analysis

Nonparametric tests were applied for statistical comparisons of gastric emptying data between different studies, since the data failed the Shapiro–Wilk test for normality. Furthermore, the sample distribution of t_{50} values tended to a certain multimodality. Thus, the Mann–Whitney test and the Kruskal–Wallis test were used when comparing two and several independent samples, respectively. The statistical analysis was performed using the SPSS program (version 15.0 SPSS Inc. Chicago, IL, USA).

Mathematical Models

The gastric emptying of pellets (GE) is defined by the portion of pellets still remaining in the stomach at a certain emptying time. Consequently, the gastric emptying profile has the following characteristics; in the beginning (time 0) the portion is 100% and it decreases to zero through time. In probabilistic terms, the gastric emptying can be defined as the probability that a pellet remains in the stomach at certain time and it could be modeled using survival (or reliability) functions. Consequently, three models were considered for describing the gastric emptying data: a lag-time exponential model, a Weibull model, and a double Weibull model (6).

The models were fitted to the obtained profiles of gastric emptying using nonlinear regression in the SPSS statistical program. The iterative Levenberg–Marquartd method was applied for the estimation of the model parameters. Standard errors of each model parameters were also obtained and the corresponding relative standard errors (RSE) of parameter estimates were calculated.

Akaike Information Criterion (AIC) was applied in order to select the most adequate model for the gastric emptying data. For this purpose, a small sample version of AIC ($\mathrm{AIC_C}$) was calculated on the basis of the following equation:

$$\mathrm{AIC_C} = n \log\left(\frac{1}{n}\sum_{i=1}^{n}\left[\mathrm{GE_{obs}}(t_i) - \mathrm{GE_{pred}}(t_i)\right]^2\right) + \frac{2pn}{(n-p-1)} \quad (1)$$

where n and p denote number of observations in each emptying profile and the number of model parameters, respectively (7). A lower value of $\mathrm{AIC_C}$ indicated a better fit.

Furthermore, model dependent estimates such as AUC and MGRT were also determined. Here, the parameter MGRT results from the application of statistical moments to the gastric emptying data (8). All calculations and graphical presentations were made in MS Excel 2003. The times for specific portions of pellets (e.g. 90%, 50%, and 10%-t_{90}, t_{50}, and t_{10}, respectively) remaining in the stomach were estimated on the basis of the model responses using MS Excel 2003 Solver. Model independent estimates of AUC and MGRT were also calculated by means of the linear trapezoidal method.

Lag-Time Exponential Model

This simple model for gastric emptying (GE) can be mathematically presented as:

$$\mathrm{GE}\,[\%] = 100e^{-k\left(t - t_{\mathrm{lag}}\right)} \quad (2)$$

Two parameters structure this model; the first order constant k (min^{-1}) and the delay in the gastric emptying t_{lag} (min). After integration of Eq. 2, the model dependent values of AUC and MGRT can be calculated as follows:

$$\mathrm{AUC_1}\,[\%\ \mathrm{min}] = \int_0^\infty \mathrm{GE}\,dt = 100t_{\mathrm{lag}} + 100k^{-1} \quad (3)$$

$$\mathrm{MGRT_1}[\mathrm{min}] = \frac{1}{\mathrm{AUC_1}}\int_0^\infty t \times \mathrm{GE}\,dt = \frac{t_{lag}^2}{2\left(t_{lag} + k^{-1}\right)} + k^{-1} \quad (4)$$

Weibull Model

Using two-parameter Weibull model; the scatter parameter η (min) and the shape parameter β, the gastric emptying can be described as:

$$\mathrm{GE} = 100e^{-\left(t/\eta\right)^{\beta}} \quad (5)$$

In fact, this model function can be transformed to a power exponential function as described in (5). Integration of Eq. (5) yields the following AUC estimate for this model:

$$AUC_2[\%\,\mathrm{min}] = \int_0^\infty GE\,dt = 100\eta\,\beta^{-1}\,\Gamma(1/\beta) \quad (6)$$

while the MGRT value can be calculated as:

$$MGRT_2[\mathrm{min}] = \frac{1}{AUC_2}\int_0^\infty t \times GE\,dt$$

$$= \frac{100\,\eta^2\,\beta^{-1}\,\Gamma(2/\beta)}{100\,\eta\,\beta^{-1}\,\Gamma(1/\beta)} = \eta\,\frac{\Gamma(2/\beta)}{\Gamma(1/\beta)} \quad (7)$$

The expression $\Gamma(\bullet)$ relates to the gamma function and was solved by means of the gamma distribution function in MS Excel 2003.

Double Weibull Model

The most complex model described here has the ability to fit the data for which emptying stops for some period of time. The model includes five parameters (η_1, η_2, β_1, β_2, and H) with the structure:

$$GE = (100 - H)\,e^{-\left(t/\eta_1\right)^{\beta_1}} + He^{-\left(t/\eta_2\right)^{\beta_2}} \quad (8)$$

This model is the sum of two Weibull models in different proportions defined by the parameter H. If the condition $\eta_1 < \eta_2$ is satisfied, then the parameter H (%) represents the fraction of pellets remaining in the stomach when the emptying has temporarily stopped.

The AUC and MGRT values can be calculated as:

$$AUC_3[\%\,\mathrm{min}] = (100 - H)\,\eta_1\,\beta_1^{-1}\,\Gamma(1/\beta_1) + H\,\eta_2\,\beta_2^{-1}\,\Gamma(1/\beta_2) \quad (9)$$

$$MGRT_3[\mathrm{min}] = \frac{1}{AUC_3}\int_0^\infty t \times GE\,dt \quad (10)$$

$$= \frac{(100 - H)\,\eta_1^2\,\beta_1^{-1}\,\Gamma(2/\beta_1) + H\,\eta_2^2\,\beta_2^{-1}\,\Gamma(2/\beta_2)}{(100 - H)\,\eta_1\,\beta_1^{-1}\,\Gamma(1/\beta_1) + H\,\eta_2\,\beta_2^{-1}\,\Gamma(1/\beta_2)}$$

RESULTS

Pellets Gastric Emptying Data

Papers studying human gastric emptying of pellets under fasting conditions in healthy subjects using the technique of gamma scintigraphy were sought using the MEDLINE database. Further information on the characteristics of the tested pellets, specific study design, and type of scintigraphic data given in these papers was carefully examined. The papers included in the evaluation of gastric emptying of pellets are shown in Table I. In these papers, (i) fasting conditions were assured; (ii) the pellets size was between 0.5 and 5 mm; and (iii) the scintigraphic data were presented graphically (as individual or mean profiles) or numerically (as t_{50} or MGRT). The papers listed in Appendix failed to fulfill the criteria above, therefore, were not included in further analyses.

From the papers in Table I some additional information was collected. Firstly, the subjects included in these studies were young and the majority was male. Secondly, the radio-labelled pellets contained an ion-exchange resin which was used as the vehicle for gamma-emitting radionuclides such as technetium (^{99m}Tc), indium (^{111}In) and samarium (^{153}Sm). Thirdly, in most cases, pellets were filled into hard gelatin capsule with fast disintegration. However, some differences among these studies regarding to the density and polymeric composition of the tested pellets, fluid intake with the administration of pellets, provision of refreshments after the administration, and subject position during the imaging were also noted. In view of this, some further selections of scintigraphic data were performed. In three studies (11,12,18), the influence of pellet density on gastric emptying was studied and only the data with a pellet density of 1.5 g/cm^3 were selected. Secondly, in two studies the influence of polycarbophil (14) and polyethylene glycol (19) on gastric emptying of pellets was investigated and only the data based on the control study were selected. Thirdly, in two studies (13,15) the influence of patient's posture on gastric emptying was examined. In most of the other studies the subjects stayed in the upright position during the period of evaluation of gastric emptying, therefore, the data obtained on the control group (upright position) were selected from these two studies.

However, none of the scintigraphic data were excluded in relation to fluid and diet intake. In five studies (10–12,18,19), pellets were administered together with 150–200 ml of orange juice, while in the others only water was used. Furthermore, in five studies, coffee was provided at 90 min post-administration (11,12,16–18). Lunch was provided approximately 3 to 4 h after the administration of the pellets in most of the studies.

Comparison Among the Studies

The parameters describing gastric emptying were used for statistical comparison of the gastric emptying kinetics among the studies reported. In six studies, t_{50} was used to represent the emptying kinetics. The distribution of t_{50} among these studies is presented in Fig. 1. The values of t_{50} significantly differed among these six studies, as suggested from the nonparametric Kruskal–Wallis test (p=0.022). These studies differed from each other according to the subjects' fluid intake (Table I). For this reason, the studies were combined into two groups; in the first group, only water was used at the time of the administration of pellets (9, 14). In the second group, pellets were administered with orange juice (11,12,18) or water (17), then at 90 min post-administration coffee was provided. The t_{50} values of these two groups were also found to be significantly different (Mann–Whitney test, p=0.016).

Table I. A list of Papers Included in the Evaluation of Gastric Emptying of Pellets Under Fasting Conditions

		Diet/fluid intake		Pellet characteristics		Scintigraphic data	
1st Author, (Ref.)	*N*	Administration	Lunch	Size (mm)	Density	Graphic (*n*)	Numeric
Yuen, (9)	6	150 ml W	At 3.3 h	1.18–1.4[b]	N/A	IND (6)	t_{50}, t_{lag}, AUC, MGRT[a]
Basit, (10)	10[c]	150 ml OJ	At 4 h	1.4–1.7[b]	N/A	IND (2)[c]	MGRT[d]
Clarke, (11)	8[c]	200 ml OJ	At 3.5 h[f]	0.5 and 4.75	1.5[e]	IND (2)[c]	t_{50}, AUC
Clarke, (12)	8	200 ml OJ	At 3.5 h[f]	1.2–1.4[b]	1.5[e]	IND (1)	t_{50}, t_{lag}, AUC
Hunter, (13)[g]	2	100 ml W	No	0.7–0.85	1.2	IND (1)	t_{lag}
Khosla, (14)	3	100 ml W	At 5 h	0.5–1.0	1.2	G (3)[h]	t_{50}[h]
Khosla, (15)[g]	5	100 ml W	N/A	0.5–1.0	1.2	G (5)	/
Hardy, (16)	4	200 ml W	At 3 h[f]	0.5–1.8	N/A	G (4)	/
Hardy, (17)	6	200 ml W	At 3 h/5 h[f]	0.5–1.8	N/A	/	t_{50}
Devereux, (18)	8	200 ml OJ	At 3.3 h[f]	1.0–1.4	1.5[e]	/	t_{50}
Basit, (19)	10	150 ml OJ	At 4 h	1.4–1.7[b]	N/A	/	MGRT[h]
Wilding, (20)	7	100 ml W	At 3 h	N/A	N/A	IND (7)	t_{50}

N number of volunteers enrolled in the fasted study, *IND* individual scintigraphic profile (number of profiles), *G* graphical presentation of mean scintigraphic data (number of volunteers), *W* water, *OJ* orange juice, *N/A* information not available, t_{50} time for 50% of pellets remaining in stomach, t_{lag} time when pellets started leaving the stomach, *AUC* area under the scintigraphic data curve, *MGRT* mean gastric residence time

[a] The data were also presented in (8)
[b] Size of the pellets without the coating
[c] Two formulations tested on the same subject
[d] Only the range of the parameter values was presented
[e] Results of pellets of only this density were taken into account in statistic analysis and modeling
[f] Coffee was also provided at 1.5 h
[g] Only the data taken at upright position of volunteers were included
[h] Only data based on the control study were enrolled

For some studies AUC data were available. The calculated median AUC values were 6,500%min (n=6), 10,100% min (n=16), and 14,900%min (n=8) from (9), (11), and (12), respectively. The Kruskal–Wallis test was performed on these data. A significant difference was found in the AUC values between these studies (p=0.0097).

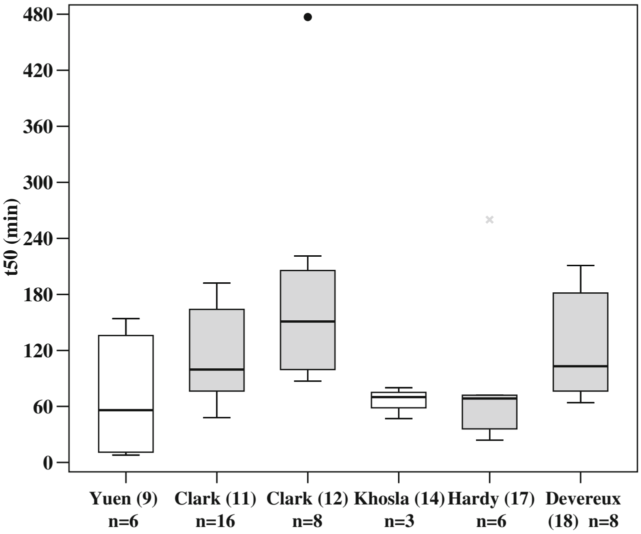

Fig. 1. Boxplot of t_{50} values for six different studies (1st Author, (Ref.), number of subjects) are presented as *white bars* (pellets were administered with water) and *gray bars* (administration with orange juice or water and at 90 min coffee was provided). Outlier (*circle*) and extreme value (*cross*) are also presented.

On the other hand, no statistically significant difference was established (Mann–Whitney test, p=0.91) when comparing the MGRT values represented in (9) and (19) with median MGRT values estimated at 47 min (n=6) and 45 min (n=10), respectively. Additionally, the values of t_{lag} in (9) and (12) did not differ significantly (Mann–Whitney test, p=0.16), despite the fact that the calculated median values of t_{lag} were 24 and 89 min in (9) and (12), respectively.

Acquired Scintigraphic Profiles

Nineteen individual scintigraphic profiles, from altogether six studies, were reconstructed (Fig. 2). In two studies scintigraphic profiles of all the subjects enrolled were reported (9,20), while in others only some selected scintigraphic profiles were reported (10–13). Additionally, three mean profiles based on three, five, and four individual profiles were obtained from (14), (15), and (16), respectively. Criteria for selection of profiles for their individual evaluation, modeling, and mean profiles calculation are described in next two sections.

Modeling of Individual Profiles

Selection of individual profiles

Altogether 12 individual profiles (profiles A to L) out of above cited 19 were included in the modeling of gastric emptying in order to describe the kinetics of individual gastric emptying of pellets (Fig. 3). All seven individual profiles from (20) were not included in the model building

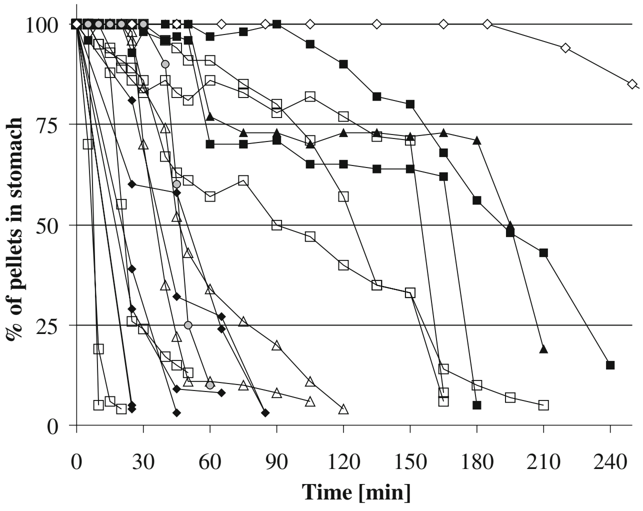

Fig. 2. Spaghetti plot of all individual scintigraphic profiles acquired from seven studies. *Open squares* six profiles from (9); *open triangles* two profiles from (10); *closed squares* two profiles from (11); *closed triangles* one profile from (12); *circles* one profile from (13); *closed diamonds* six profiles from (20); *open diamonds* the profile of a subject in gastric stasis from (20).

process as the reported data were mostly sparse (infrequent data imaging). The profiles from (9), which were obtained after administration of pellets to subject NA, KI, SL, JE, VK, and JA were renumbered as profile A, B, C, D, E, and F, respectively. The two scintigraphic profiles graphically presented in (10) are from the same subject but for two different formulations; the same was observed in (11). In the former study (10) small intestine release (profile G) and colon release pellets (profile H) were tested, while the scintigraphic profiles in the latter study (11) represent emptying pellets of two different sizes; 0.5 mm (profile I) and 4.75 mm (profile J) and of density 1.5 g/cm^3. In both cases the differences in physico-chemical properties of the tested formulations were considered to have a negligible effect on gastric emptying of pellets, as concluded in these studies (10,11). Two additional individual scintigraphic profiles, profiles K and L, were obtained from (12) and (13), respectively.

Modeling of individual profiles

Three mathematical models were fitted to the 12 above cited individual scintigraphic profiles (Fig. 3). Values of the estimated parameters together with their RSEs and the calculated AIC_C values are shown in Table II. In three cases (profiles A, B, and L) the number of data observed in the emptying phase was too small for fitting the double Weibull model.

The kinetics of six gastric emptying profiles (from A to F) from (9) was fully numerically described by the following parameters: the time for 90%, 50%, and 10% of pellets remaining in the stomach (t_{90}, t_{50} and t_{10}, respectively), AUC, and MGRT (8,9). The same parameters were also calculated on the basis of double Weibull model responses of these profiles (Table III). As in this model no t_{lag} is involved, t_{90} was used as an approximation for the t_{lag} reported in (9). Similarly, t_{10} was used as an approximation for the time point when emptying was recognized to be complete, as reported in (9). The gastric emptying parameters as reported in (8) and (9) were compared to the same parameters calculated from the double Weibull model (Table III). The differences of the model based calculations of t_{50}, AUC and MGRT from the reported values were less than 10% with the exception of the two fast profiles A and B.

Evaluation of Other Profiles

Profiles from (20) were excluded from the evaluation of modeling of individual profiles due to sparse data points. However, to estimate additional data points in order to obtain the gastric emptying profiles dense enough for calculation of mean gastric emptying profiles these data were additionally modeled. Among seven profiles, one profile (profile P) was separately treated as the subject was in almost total gastric stasis (subject 7 in (20)). To the other six profiles, the double Weibull model was fitted whenever possible; to the profiles with number of data points less than 5 the Weibull model was fitted instead.

The lag-time exponential, Weibull, and double Weibull models were fitted to mean scintigraphic profiles M, N, and O from (14), (15), and (16), respectively (Fig. 4). According to AIC_C values the double Weibull model fitted the data better.

Determination of Mean Gastric Emptying of Pellets

All 19 individual gastric emptying profiles were combined into two groups; seven individual profiles indicated slow emptying of pellets from the stomach (profiles D, E, F, I, J, K, and P), whereas the other 12 individual profiles (profiles A, B, C, G, H, L, and six individual profiles from (20)) indicated fast emptying. Separately for each group the average of gastric emptying at each time point was calculated and mean fast and mean slow gastric emptying profiles of pellets were generated (Fig. 5).

The overall mean gastric emptying profile was estimated. For this purpose, 16 individual profiles out of 19 were averaged at each time point, generating mean individual profile. Profiles I, J, and L were excluded from this calculation, as these profiles did not represent typical profiles in the original study. Additionally, the three mean scintigraphic profiles (M, N, and O) were included in the calculation. A weighted average of the three mean scintigraphic profiles and the mean individual profile (n=16) was calculated at each time point, resulting in the overall mean gastric emptying profile (n=28). Due to the exclusion of profile J, the interval of pellets size included in overall mean gastric emptying profile generation narrowed to 0.5–1.8 mm.

The double Weibull and Weibull models were fitted to overall, fast, and slow mean gastric emptying profiles (Fig. 5). Models parameters together with some additional parameters are presented in Table IV.

DISCUSSION

In this study, a review of the evaluation of gastric emptying of pellets based on imaging by gamma scintigraphy

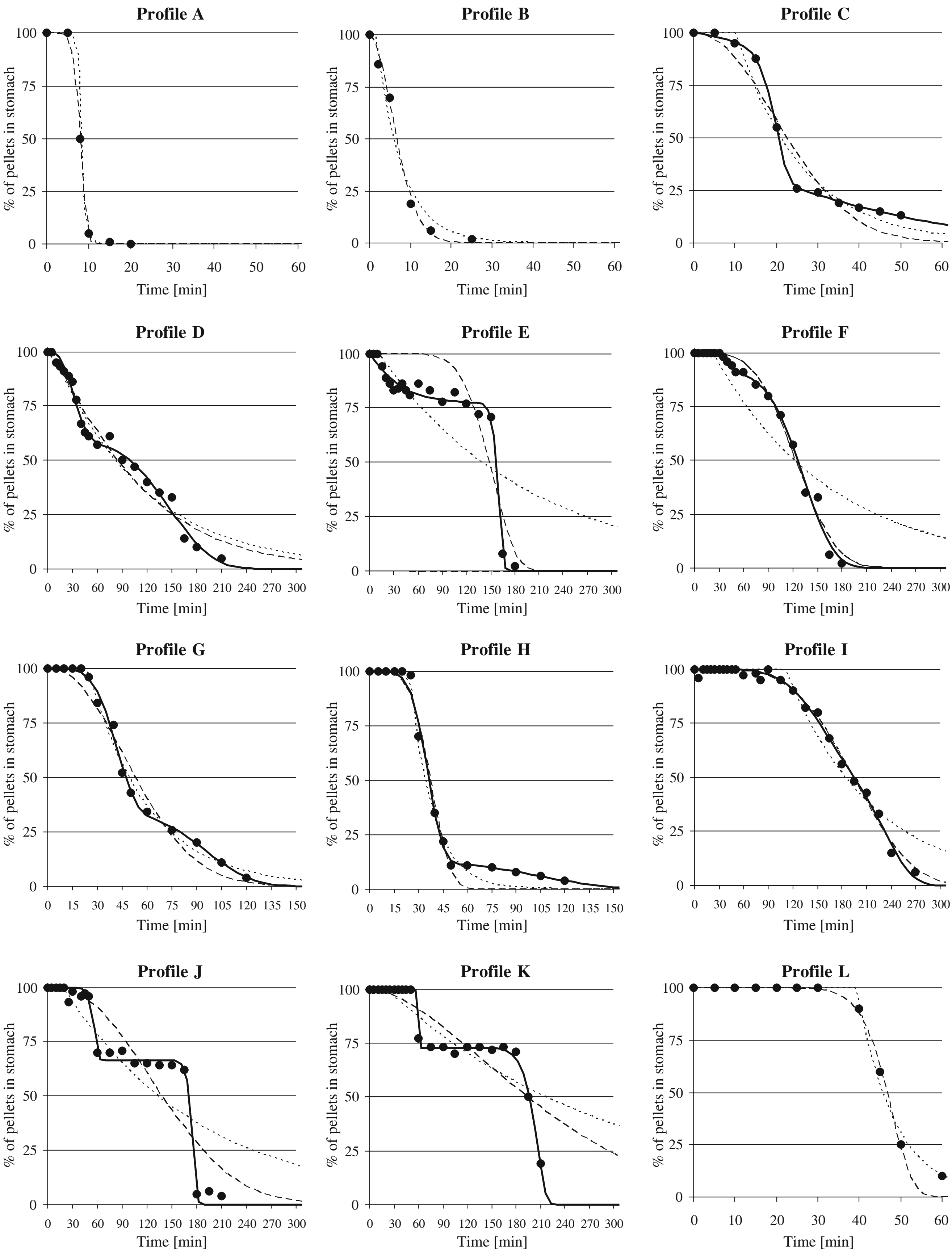

Fig. 3. Lag-time exponential model (*dotted line*), Weibull model (*dashed line*) and double Weibull model (*solid line*, not obtained for profiles A, B, and L) fitted to individual scintigraphic profiles from A to L (*filled circles*) as obtained from (9–13).

Table II. Model Parameter Estimates (RSE in %) of Different Models Fitted to Individual Scintigraphic Profiles from (9–13)

Model parameters	Profile A	Profile B	Profile C	Profile D	Profile E	Profile F
Lag-time exponential model						
t_{lag} (min)	7.4 (0.81)	1.4 (48)	10.2 (11)	5.7 (50)	10.5 (>100)	22.0 (28)
k (min^{-1})	1.15 (8.6)	0.159 (22)	0.0641 (12)	0.00917 (6.5)	0.00543 (20)	0.00698 (15)
AIC_C	1.8	31.1	47.1	79.5	119	110
Weibull model						
η (min)	8.45 (0.73)	7.99 (5.3)	26.8 (5.8)	111 (4.5)	157 (3.6)	136 (1.5)
β	6.97 (8.4)	1.84 (12)	2.08 (17)	1.18 (7.4)	6.47 (30)	3.61 (7.1)
AIC_C	12.9	23.4	52.4	77.7	109	58
Double Weibull model						
η_1 (min)	/	/	20.4 (0.71)	36.6 (6.7)	36.1 (27)	44.4 (12.9)
β_1	/	/	8.04 (14)	2.91 (17)	1.08 (29)	6.96 (89)
H (%)	/	/	39.3 (6.7)	58.5 (5.8)	77.1 (3.5)	91.1 (3.1)
η_2 (min)	/	/	45.8 (6.0)	159 (2.7)	161 (0.6)	141 (1.4)
β_2	/	/	1.45 (7.4)	3.94 (18)	33.8 (19)	4.66 (9.8)
AIC_C	/	/	14.4	59.3	55.0	54.2
	Profile G	Profile H	Profile I	Profile J	Profile K	Profile L
Lag-time exponential model						
t_{lag} (min)	24.0 (5.7)	24.7 (2.6)	111 (5.7)	18.6 (44)	19.9 (44)	39.2 (2.6)
k (min^{-1})	0.0283 (7.3)	0.0707 (8.6)	0.00951 (13)	0.00607 (16)	0.00352 (15)	0.109 (24)
AIC_C	42.3	43.4	94.2	117	105	33.9
Weibull model						
η (min)	62.5 (4.1)	40.5 (2.4)	212 (0.7)	162 (5.9)	241 (12)	48.3 (0.3)
β	2.13 (11)	4.35 (14)	3.93 (3.8)	2.28 (20)	1.66 (21)	10.5 (4.1)
AIC_C	59.9	57.7	47.4	109	103	9.7
Double Weibull model						
η_1 (min)	43.9 (3.7)	37.7 (2.8)	159 (13)	56.8 (2.6)	59.6 (>100)	/
β_1	4.57 (12.6)	5.17 (12)	4.96 (17)	14.1 (29)	90.4 (>100)	/
H (%)	34.5 (20.9)	12.4 (55)	57.6 (38)	66.5 (2.0)	72.8 (0.7)	/
η_2 (min)	102 (6.5)	115 (21)	237 (5.1)	176 (0.7)	206 (0.2)	/
β_2	4.56 (44.2)	3.46 (>100)	8.15 (42)	41.2 (23)	19.1 (5.5)	/
AIC_C	41.9	49.2	50.6	58.2	17.0	/

AIC_C Akaike Information Criteria, *>100* value greater than 100%

was focused in order to obtain the mean gastric emptying of pellets. With this technique decay of the initial gamma emitting activity of the pellets in the stomach region with time can be recorded. The proportion of initial gamma emitting activity is equal to the proportion of pellets remaining in the stomach. Thus, the scintigraphic profiles provided from the selected studies also represent the gastric emptying pattern of the pellets. However, whether the accuracy of scintigraphic imaging in different studies was comparable is debatable, as these studies were performed over a period of 20 years in

Table III. Emptying Parameters Calculated from Double Weibull Model (M) Compared to the Emptying Parameters of the Same Subjects as Previously Reported in (8) or (9)

	t_{90} (min)		t_{50} (min)		t_{10} (min)		AUC (%min)		MGRT (min)	
Profile	(9)	M	(9)	M	(9)	M	(8)	M	(8)	M
A[a]	4	6.1	8	8	18	9.5	839	790	6.5	4.1
B[a]	7	2.3	11	6.5	16	12.6	1,063	709	6.2	4.7
C	20	14	24	20.5	41	56.8	2,849	2,798	17.9	22.1
D	27	23	88	99.4	184	183.8	10,147	9,781	78.2	69.5
E	120	21.6	154	157.1	215	164.5	13,229	13,022	79.8	76.4
F	95	52.7	136	170.6	187	178.6	12,793	12,090	75.7	78.7

t_{90} time for 90% of pellets remaining in the stomach, *t_{50}* time for 50% of pellets remaining in the stomach, *t_{10}* time for 10% of pellets remaining in the stomach, *AUC* area under the scintigraphic data curve, *MGRT* mean gastric residence time
[a] The parameters were calculated on the basis of the Weibull model due to sparse emptying data.

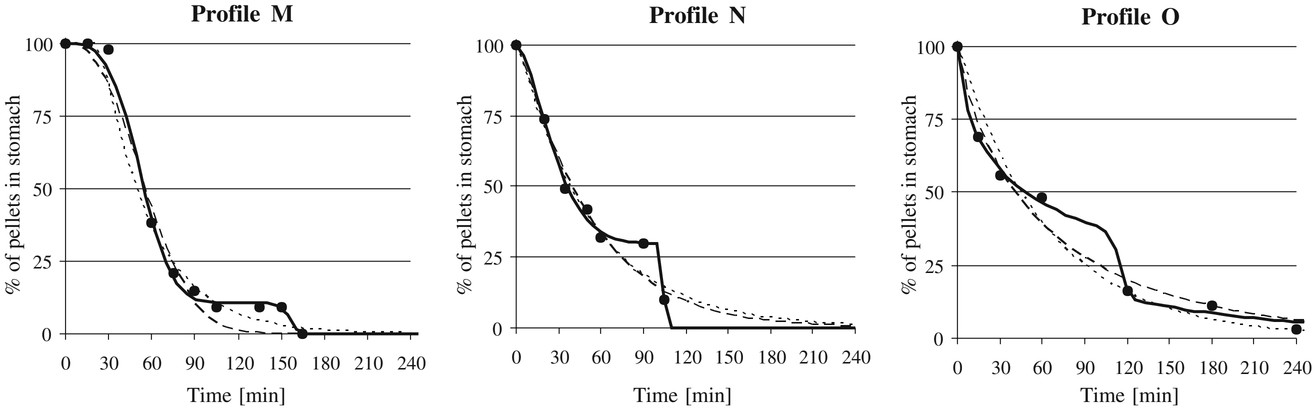

Fig. 4. Lag-time exponential model (*dotted line*), Weibull model (*dashed line*) and double Weibull model (*solid line*) fitted to mean profiles from M to O (*filled circles*) as obtained from (14–16).

which the gamma scintigraphic technique was probably improved remarkably. Furthermore, the studies differed according to their study design. The size and density of the tested pellets, liquids intake at the time of administration of the pellets, provision of refreshments after the administration, and the subjects' position during imaging were examined in each paper. The diversity amongst the studies may not have had a negligible effect on the variability of the profiles obtained. Due to this fact, the above mentioned criteria had to be necessarily defined, but only a few studies met these criteria. Accordingly, the number of scintigraphic profiles obtained was relatively low. Thus, a study of gastric emptying of pellets under fasting conditions, from which consistent gastric emptying data would be obtained, should be performed on large number of subjects.

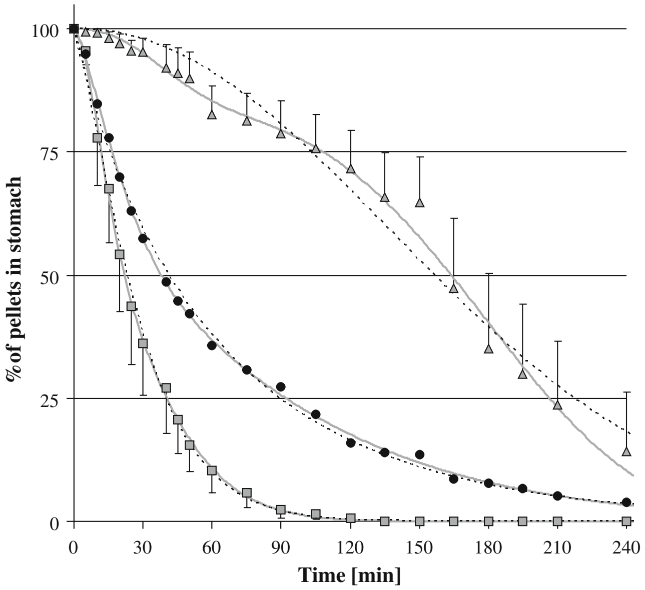

Fig. 5. Overall mean gastric emptying profile of pellets (*circles*, $n=28$) and mean gastric emptying of fast (*squares*, $n=12$) and slow (*triangles*, $n=7$) profiles together with standard errors of mean. *Solid lines* double Weibull model, *dashed line* Weibull model.

The design of such a study should include the following guidelines: pellets should be administered with water, no refreshments should be provided for a period of at least two MMC cycles (at least 4 h) and frequent data collection should be assured over a longer period of time.

A large diversity in the design of the refreshments provided at the time of administration and during imaging was observed (Table I). It is already known that food prolongs the gastric emptying of pellets. However, the beverage intake could also affect gastric emptying, especially if the beverage had high caloric values. It was reported (21) that under fasting conditions the gastric emptying (t_{50}) of 500 mL of glucose solutions in concentrations of 5.5% (110 kcal) and 11.4% (228 kcal) was 29.7 and 64.8 min, respectively ($n=10$). In the same study, the reported t_{50} values were significantly longer than 14.6 min, which was the t_{50} determined for 500 mL of water ($n=10$). Although the volume and the caloric values of the beverages administered in the studies were much lower, influence on the gastric emptying of pellets cannot be excluded. In fact, from the data presented in Fig. 1 and the fact that statistically significant differences were noted among these studies, it can be concluded that application of pellets with orange juice and coffee intake at 90 min after the application may prolong the gastric emptying of pellets. However, such conclusions are speculative due to the lack of detailed information regarding the study protocols.

Nevertheless, from the acquired scintigraphic profiles the following characteristics of the gastric emptying profile can be observed. Firstly, a large variability in gastric emptying kinetics was detected, with emptying time varying from approximately 15 min to more than 3 h (Fig. 2). Moreover, in 12 of 19 individual profiles emptying was completed in less than 2 h, while in most of the other profiles the majority of pellets were still in the stomach after 2 h. Secondly, from the profiles in Fig. 3, different patterns of emptying were observed. In three profiles pellets were emptied almost immediately and rapidly (profiles A, B, and C in Fig. 3). In three profiles a short delay (t_{lag} less than 30 min) occurred, followed by a rapid emptying period (profiles G, H, and L in Fig. 3). On the other hand, for the rest of the profiles pellets were emptied more slowly, resulting in longer t_{50} and MGRT

Table IV. Parameter Estimates (RSE in %) of the Double Weibull and the Weibull Models Fitted to Mean Gastric Emptying (GE) Profiles

Parameter	Mean fast GE profile (n=12)		Overall mean GE profile (n=28)		Mean slow GE profile (n=7)	
	Double Weibull	Weibull	Double Weibull	Weibull	Double Weibull	Weibull
η_1 (min)	14.8 (14)	/	22.1 (10)	/	45.8 (19)	/
β_1	1.91 (30)	/	1.42 (12)	/	2.34 (35)	/
H (%)	68.2 (41)	/	62.5 (21)	/	84.1 (3.9)	/
η_2 (min)	40.2 (26)	/	98.9 (18)	/	196 (1.6)	/
β_2	1.53 (28)	/	1.21 (17)	/	3.66 (11)	/
η (min)	/	30.7 (1.3)	/	61.9 (1.7)	/	186 (2.4)
β	/	1.26 (2.6)	/	0.895 (2.4)	/	2.11 (7.3)
AIC_C	11.1	22.4	3.34	32.3	50.6	69.8
t_{90} (min)	6.5	5.1	7.5	5.0	45	64
t_{50} (min)	22	23	38	41	164	156
t_{10} (min)	62	60	163	157	241	277
AUC (%min)	2,890	2,860	6,560	6,530	15,500	16,500
MGRT (min)	24	23	71	74	94	103

AUC area under the scintigraphic data curve, *MGRT* mean gastric residence time, t_{90} time for 90% of pellets remaining in the stomach, t_{50} time for 50% of pellets remaining in the stomach, t_{10} time for 10% of pellets remaining in the stomach

values. In these profiles two different patterns were distinguished. In the first, a delay in emptying of pellets longer than 30 min followed by a slow emptying period (profiles F and I in Fig. 3) was noted. In the second, a certain interruption of emptying of pellets occurred, resulting in overall prolonged gastric emptying of pellets (profiles D, E, J, and K in Fig. 3). In these cases the pellets seemed to empty as two relatively brief boluses.

According to the graphical comparisons, the double Weibull model appeared to fit adequately all individual gastric emptying profiles, especially in the case of any kind of interruption in emptying of pellets (Fig. 3). Elashoff *et al.* (5) pointed out that the model used for analyzing gastric emptying data should have as few parameters as possible. However, the number of model parameters is included in the calculation of AIC_C and in most cases the AIC_C values were the lowest for the double Weibull model. Thus, it can be concluded that the double Weibull model fitted best to the individual gastric emptying profiles, despite the fact that the data with too low number of observations in the emptying period could not be fitted with this model. Some high values for RSE of the model parameter estimates were observed (Table II). Sparse data points in the emptying period could be the reason for the high RSEs of the parameter estimates.

By fitting the double Weibull model to individual scintigraphic profiles, some further characteristics in emptying pattern can be revealed. The parameter H represents the portion of pellets remaining in the stomach when interruption of emptying occurred. However, this parameter has little importance when it is estimated to be above 90% or below 10%, as the scintigraphic data in this region are hard to evaluate adequately (e.g. high oscillation of the gamma emitting activity). The duration of this interruption can be assigned as the difference between η_2 and η_1. Moreover, in the four profiles D, E, J, and K this difference was between 2.0 and 2.5 h (Table II), which corresponds to the length of one MMC cycle. The emptying pattern for these profiles indicates that the emptying of the majority of the pellets occurs during the short periods of intense contractions (phase III) of two consecutive MMC cycles, while much slower and diminishing emptying occurs during the other phases of the MMC cycles. Thus, for the profiles with such emptying pattern, the difference in η_1 and η_2 indicates the time period between the two consecutive MMC cycles, which gives an additional applicative value to the double Weibull model.

Marked intra- and inter-individual variability in the gastric emptying process has already been reported and one of the possible influences mentioned by Yuen *et al.* (9) was also the presence of slow and fast emptiers which was previously noted by other authors. In this view, mean slow and fast gastric emptying profiles were also calculated. Marked difference between these two groups of profiles was observed (Fig. 5).

Despite this, the overall mean gastric emptying profile calculated in this study was still considered to adequately represent gastric emptying of pellets under fasting conditions. This profile was calculated in order to be used in the development of *in vitro* dissolution testing model. Therefore, it had to simulate well the average situation in the stomach after administration of pellets. Thus, all available data, individual and mean, were included except the individual profiles, which were presented in particular studies only as examples and were not typical profiles of the studies. This resulted in narrower size interval of pellets included in overall mean gastric emptying profile development, which was finally 0.5–1.8 mm.

Although the AIC_C values for fitting double Weibull model to overall mean GE profile were lower than for Weibull model (Table IV), the latter model still sufficiently well describes the majority of the emptying process, when comparing the values such as AUC, MGRT, t_{90}, and t_{50}, and t_{10}. Additionally, as mentioned before, the double Weibull model fitted better to individual gastric emptying profiles where emptying occurred as a series of boluses. However, by

calculation of mean profiles the process of emptying in boluses became diminished, which additionally indicated that the Weibull model was adequate enough to describe the mean gastric emptying profile.

Furthermore, the overall mean gastric emptying described by the Weibull model as:

$$\mathrm{GE}(t) = 100\, e^{-\left(t[\min]/61.9\right)^{0.895}} \quad (11)$$

could be applied in the determination of experimental conditions of *in vitro* dissolution tests; i.e. residence times of portions of pellets in acidic medium, for pellet formulations for which pH dependent dissolution profiles were observed.

CONCLUSIONS

From the studies selected, scintigraphic profiles representing the gastric emptying of pellets under fasting conditions in healthy subjects were obtained and different patterns of gastric emptying were observed, with the emptying time varying from approximately 15 min to more than 3 h. Three mathematical models were fitted to the gastric emptying profiles. The best model for fitting the individual gastric emptying profiles was the double Weibull model, especially in the case of interruption of emptying of pellets as observed in some individual gastric emptying profiles. In these cases, the double Weibull model has some additional applicative value, meaning that the difference in η_1 and η_2 could serve as a parameter indicating the time period between the two consecutive MMC cycles. On the other hand, by calculation of the mean gastric emptying profile, the process of emptying in boluses is diminished and the overall mean gastric emptying profile is also adequately described by the Weibull model. Additionally, due to its simplicity, which can be viewed as an advantage over the double Weibull model, the Weibull model describing mean gastric emptying of pellets could be successfully applied in the design of *in vitro* dissolution experiments.

APPENDIX

Table V. A List of Papers Studying the Human Gastric Emptying of Pellets Under Fasting Conditions not Included in the Evaluation Due to Reasons Stated under *Comments*

		Diet/fluid intake		Pellet characteristics			
1st Author, (Ref.)	*N*	Administ.	Lunch	Size (mm)	Density	Scintigraphic data (n)	Comments
Davis, (22)	8	N/A	N/A	N/A	N/A	G (8)	The same data as in (20)
Davis, (23)[a]	20	100 ml W	at 3–4 h	0.3 (P1)	N/A	t_{50}	P1 (*n*=4): pellet size <0.5 mm
Hardy, (24)	8	200 ml W	at 4 h	0.8–1.2	N/A	G (median)	Scintigraphic data not applicable
Wilding, (25)	7	100 ml W	at 4 h	/	/	G (7), t_{50} (7)	Tablets disintegrating into pellets
Kenyon (26)	8	150 ml W	at 4 h	/	/	G (8), t_{50} (8)	Tablets disintegrating into pellets
Davis, (27)	6	100 ml W	*Ad libitum*	0.8–1.1	1.2	G (6), t_{50} (6)	Fasting conditions not assured
Davis, (28)	6	N/A	N/A	0.3–1.2	N/A	G (6)	Pellet size <0.5 mm
Digenis, (29)	6	N/A	N/A	0.1–0.4	N/A	IND (1)	Pellet size <0.5 mm
Digenis, (30)	7	200 ml W	at 8 h	N/A	N/A	graphic	Scintigraphic data not applicable
Graffner, (31)	8	100 ml W	at 4 h	1.0–1.4	N/A	RTS (8)	Scintigraphic data not applicable
Christensen, (32)	8	300 ml W	*Ad libitum*	0.7–1.4	1.8	G (8), t_{50} (8)	Fasting conditions not assured
Sugito, (33)	4	200 ml W	no	8×4	1.33	G (4), t_{50} (4)	Size >5 mm
Beten, (34)	6	150 ml W	N/A	0.1–0.5	1.36	t_{50} (6)	Pellet size <0.5 mm
Hunter, (35)	11	100 ml W	no	<0.1	1.2	IND (5)	Particle size <0.5 mm
Wilding, (36)	8	150 ml W	4 h	mini tablets		t_{50} (8)	Size >5 mm
Wilding, (37)	8	200 ml W	4 h	microgranules		G (8), t_{50} (8)	Particle size <0.5 mm
Wilding, (38)	12	200 ml W	4 h	micropellets		t_{50} (12)	Particle size <0.5 mm
Brunner, (39)	14	200 ml W	N/A	N/A		t_{50} (mean)	Formulation properties unknown
Podczeck, (40)	8	50 mL W	at 3.5 h	mini tablets		t_{lag} (8), t_{FE} (8)	Emptying data not applicable

N number of volunteers enrolled in the fasted study, *IND* individual scintigraphic profile (number of profiles), *G* graphical presentation of mean scintigraphic data (number of volunteers), *W* water, *N/A* information not available, t_{50} time for 50% of pellets remaining in stomach, t_{lag} time of first emptying, t_{FE} time of final emptying, *RTS* stomach resistance time.

[a] Four studies (named P1–P4) under fasting conditions are presented, P2 data are the same as in (17), in P3 and P4 subjects had constipation or diarrhea.

REFERENCES

1. I. R. Wilding, A. J. Coupe, and S. S. Davis. The role of gamma-scintigraphy in oral drug delivery. *Adv. Drug. Deliv. Rev.* **46**:103–124 (2001). doi:10.1016/S0169-409X(00)00135-6.
2. A. J. Moës. Gastroretentive dosage forms. *Crit. Rev. Ther. Drug. Carrier. Syst.* **10**:143–195 (1993).
3. N. Follonier, and E. Doelker. Biopharmaceutical comparison of oral multiple-unit and single-unit sustained-release dosage forms. *S.T.P. Pharma. Sci.* **2**:141–158 (1992).
4. L. Shargel, S. Wu-Pong, and A. B. C. Yu. Physiologic factors related to drug absorption. In: *Applied biopharmaceutics and pharmacokinetics,* 5th ed. McGraw-Hill, New York, 2005, pp. 371–409.
5. J. D. Elashoff, T. J. Reedy, and J. H. Meyer. Analysis of gastric emptying data. *Gastroenterology.* **83**:1306–1312 (1982).
6. D. N. P. Murthy, M. Xie, and R. Jiang. *Weibull models*. Wiley, Hoboken, New Jersey, 2004, pp. 106–109.
7. K. P. Burnham, and D. R. Anderson. Multimodel inference—understanding AIC and BIC in model selection. *Sociol. Method. Res.* **33**:261–304 (2004). doi:10.1177/0049124104268644.
8. F. Podczeck, J. M. Newton, and K. H. Yuen. The description of the gastrointestinal transit of pellets assessed by gamma scintigraphy using statistical moments. *Pharm. Res.* **12**:376–379 (1995). doi:10.1023/A:1016200501563.
9. K. H. Yuen, A. A. Deshmukh, J. M. Newton, M. Short, and R. Melchor. Gastrointestinal transit and absorption of theophylline from a multiparticulate controlled-release formulation. *Int. J. Pharm.* **97**:61–77 (1993). doi:10.1016/0378-5173(93)90127-2.
10. A. W. Basit, F. Podczeck, J. M. Newton, W. A. Waddington, P. J. Ell, and L. F. Lacey. The use of formulation technology to assess regional gastrointestinal drug absorption in humans. *Eur. J. Pharm. Sci.* **21**:179–189 (2004). doi:10.1016/j.ejps.2003.10.003.
11. G. M. Clarke, J. M. Newton, and M. D. Short. Gastrointestinal transit of pellets of differing size and density. *Int. J. Pharm.* **100**:81–92 (1993). doi:10.1016/0378-5173(93)90078-T.
12. G. M. Clarke, J. M. Newton, and M. B. Short. Comparative gastrointestinal transit of pellet systems of varying density. *Int. J. Pharm.* **114**:1–11 (1995). doi:10.1016/0378-5173(94)00200-O.
13. E. Hunter, J. T. Fell, and H. Sharma. The gastric-emptying of pellets contained in hard gelatin capsules. *Drug Dev. Ind. Pharm.* **8**:751–757 (1982). doi:10.3109/03639048209042700.
14. R. Khosla, and S. S. Davis. The effect of polycarbophil on the gastric emptying of pellets. *J. Pharm. Pharmacol.* **39**:47–49 (1987).
15. R. Khosla, and S. S. Davis. The gastric emptying of pellets in supine volunteers. *J. Pharm. Pharmacol.* **38**:P10 (1986).
16. J. G. Hardy, and A. C. Perkins. Validity of the geometric mean correction in the quantification of whole bowel transit. *Nucl. Med. Commun.* **6**:217–224 (1985). doi:10.1097/00006231-198504000-00005.
17. J. G. Hardy, C. G. Wilson, and E. Wood. Drug delivery to the proximal colon. *J. Pharm. Pharmacol.* **37**:874–877 (1985).
18. J. E. Devereux, J. M. Newton, and M. B. Short. The influence of density on the gastrointestinal transit of pellets. *J. Pharm. Pharmacol.* **42**:500–501 (1990).
19. A. W. Basit, J. M. Newton, M. D. Short, W. A. Waddington, P. J. Ell, and L. F. Lacey. The effect of polyethylene glycol 400 on gastrointestinal transit: implications for the formulation of poorly-water soluble drugs. *Pharm. Res.* **18**:1146–1150 (2001). doi:10.1023/A:1010927026837.
20. I. R. Wilding, J. G. Hardy, M. Maccari, V. Ravelli, and S. S. Davis. Scintigraphic and pharmacokinetic assessment of a multiparticulate sustained-release formulation of diltiazem. *Int. J. Pharm.* **76**:133–143 (1991). doi:10.1016/0378-5173(91)90351-N.
21. A. Franke, S. Teyssen, H. Harder, and M. V. Singer. Effect of ethanol and some alcoholic beverages on gastric emptying in humans. *Scand. J. Gastroentero.* **39**:638–644 (2004). doi:10.1080/00365520410005009.
22. S. S. Davis, J. G. Hardy, S. P. Newman, and I. R. Wilding. Gamma scintigraphy in the evaluation of pharmaceutical dosage forms. *Eur. J. Nucl. Med.* **19**:971–986 (1992). doi:10.1007/BF00175865.
23. S. S. Davis, J. G. Hardy, and J. W. Fara. Transit of pharmaceutical dosage forms through the small-intestine. *Gut.* **27**:886–892 (1986). doi:10.1136/gut.27.8.886.
24. J. G. Hardy, G. L. Lamont, D. F. Evans, A. K. Haga, and O. N. Gamst. Evaluation of an enteric-coated naproxen pellet formulation. *Aliment. Pharmacol. Ther.* **5**:69–75 (1991).
25. I. R. Wilding, S. S. Davis, R. A. Sparrow, J. R. Bloor, G. Hayes, and G. T. Ward. The effect of food on the *in vivo* behaviour of a novel sustained release formulation of tiaprofenic acid. *Int. J. Pharm.* **83**:155–161 (1992). doi:10.1016/0378-5173(82)90018-7.
26. C. J. Kenyon, G. Hooper, D. Tierney, J. Butler, J. Devane, and I. R. Wilding. The effect of food on the gastrointestinal transit and systemic absorption of naproxen from a novel sustained-release formulation. *J. Control. Release* **34**:31–36 (1995). doi:10.1016/0168-3659(94)00118-E.
27. S. S. Davis, J. G. Hardy, M. J. Taylor, D. R. Whalley, and C. G. Wilson. A comparative-study of the gastrointestinal transit of a pellet and tablet formulation. *Int. J. Pharm.* **21**:167–177 (1984). doi:10.1016/0378-5173(84)90091-7.
28. S. S. Davis. The design and evaluation of controlled release systems for the gastrointestinal tract. *J. Control. Release* **2**:27–38 (1985). doi:10.1016/0168-3659(85)90030-6.
29. G. A. Digenis, E. P. Sandefer, A. F. Parr, R. Beihn, C. McClain, B. M. Scheinthal, I. Ghebre-Sellassie, U. Iyer, R. U. Nesbitt, and E. Randinitis. Gastrointestinal behavior of orally-administered radiolabeled erythromycin pellets in man as determined by gamma scintigraphy. *J. Clin. Pharmacol.* **30**:621–631 (1990).
30. G. A. Digenis, E. P. Sandefer, R. C. Page, and W. J. Doll. Gamma scintigraphy: an evolving technology in pharmaceutical formulation development—Part 1. *Pharm. Sci. Technol. To.* **1**:100–107 (1998). doi:10.1016/S1461-5347(98)00032-7.
31. C. Graffner, Z. Wagner, M. I. Nilsson, and E. Widerlov. Plasma-concentrations of remoxipride and the gastrointestinal transit of In-111-marked extended-release coated spheres. *Pharm. Res.* **7**:54–58 (1990). doi:10.1023/A:1015835609333.
32. F. N. Christensen, S. S. Davis, J. G. Hardy, M. J. Taylor, D. R. Whalley, and C. G. Wilson. The use of gamma-scintigraphy to follow the gastrointestinal transit of pharmaceutical formulations. *J. Pharm. Pharmacol.* **37**:91–95 (1985).
33. K. Sugito, H. Ogata, H. Goto, M. Noguchi, T. Kogure, M. Takano, Y. Maruyama, and Y. Sasaki. Gastrointestinal transit of nondisintegrating solid formulations in humans. *Int. J. Pharm.* **60**:89–97 (1990). doi:10.1016/0378-5173(90)90294-E.
34. D. B. Beten, B. Vangansbeke, A. Schoutens, and A. J. Moës. Evaluation of the gastric behavior of coevaporate particles under fasting and nonfasting conditions. *Int. J. Pharm.* **123**:145–147 (1995). doi:10.1016/0378-5173(95)00087-Y.
35. E. Hunter, J. T. Fell, and H. Sharma. The gastric-emptying of hard gelatin capsules. *Int. J. Pharm.* **17**:59–64 (1983). doi:10.1016/0378-5173(83)90018-2.
36. I. R. Wilding, S. S. Davis, R. A. Sparrow, J. A. Ziemniak, and D. L. Heald. Pharmacoscintigraphic evaluation of a modified release Geomatrix(R) diltiazem formulation. *J. Control. Release* **33**:89–97 (1995). doi:10.1016/0168-3659(94)00066-4.
37. I. R. Wilding, C. J. Kenyon, and G. Hooper. Gastrointestinal spread of oral prolonged-release mesalazine microgranules (Pentasa) dosed as either tablets or sachet. *Aliment. Pharmacol. Ther.* **14**:163–169 (2000). doi:10.1046/j.1365-2036.2000.00696.x.
38. I. R. Wilding, C. Behrens, S. J. Tardif, H. Wray, P. Bias, and W. Albrecht. Combined scintigraphic and pharmacokinetic investigation of enteric-coated mesalazine micropellets in healthy subjects. *Aliment. Pharmacol. Ther.* **17**:1153–1162 (2003). doi:10.1046/j.1365-2036.2003.01558.x.
39. M. Brunner, R. Greinwald, K. Kletter, H. Kvaternik, M. E. Corrado, H. G. Eichler, and M. Müller. Gastrointestinal transit and release of 5-aminosalicylic acid from Sm-153-labelled mesalazine pellets *vs.* tablets in male healthy volunteers. *Aliment. Pharmacol. Ther.* **17**:1163–1169 (2003). doi:10.1046/j.1365-2036.2003.01564.x.
40. F. Podczeck, N. Course, J. M. Newton, and M. B. Short. Gastrointestinal transit of model mini-tablet controlled release oral dosage forms in fasted human volunteers. *J. Pharm. Pharmacol.* **59**:941–945 (2007). doi:10.1211/jpp.59.7.0005.
